# A polymorphism and expression levels of *SCD* and their relationships to production traits in a Japanese farmed emu population

**DOI:** 10.1371/journal.pone.0352695

**Published:** 2026-06-25

**Authors:** Yuichi Koshiishi, Suzuka Moriya, Yuki Ishida, Hiromitsu Ochiai, Kenta Wada

**Affiliations:** 1 International Institute for Zoonosis Control, Hokkaido University, Sapporo, Hokkaido, Japan; 2 Faculty of Bioindustry, Tokyo University of Agriculture, Abashiri, Hokkaido, Japan; 3 Graduate School of Bioindustry, Tokyo University of Agriculture, Abashiri, Hokkaido, Japan; Hamadan University of Medical Sciences, IRAN, ISLAMIC REPUBLIC OF

## Abstract

The emu is a novel poultry species producing meat, eggs, and fat. In particular, emu oil derived from the fat is considered the most important product of the emu owing to its anti-inflammatory and anti-melanin production. Meanwhile, there are few reports on the genetic improvement of economic traits in emus. In this study, we discovered a non-synonymous substitution (c.267A > C, p.Leu89Phe) in the gene encoding stearoyl-CoA desaturase (SCD), a key enzyme involved in unsaturated fatty acid synthesis, in a Japanese farmed emu population. Although fat yields (fat weight per body weight) in males with AA and AC genotypes of *SCD* were higher than those in their female counterparts, significant intra-sex variations were not detected across genotypes in our study population. Moreover, fat melting points and fatty acid composition did not significantly vary between *SCD* genotypes. These results suggest that the *SCD* c.267A > C polymorphism does not affect fat production traits in emus. However, the expression levels of *SCD* transcripts were negatively correlated with fat content (P < 0.01) and positively correlated with meat yield (P < 0.05) in emus.

## Introduction

The emu (*Dromaius novaehollandiae*), which originated from Australia, is the second-largest bird species and belongs to the ratite family. In the 1970s, Australia made an initial attempt to utilize emus for leather production. Subsequently, the industrial application for meat, egg, and oil production using emus expanded to other countries, such as the USA, Europe, Asia, and Africa [1]. Among their products, oil purified from adipose tissue is highly valued as a raw material for cosmetic and skincare products because it has been reported to exhibit anti-inflammatory [2–4] and anti-melanogenic effects [5]. Therefore, the genetic improvement of oil production-related traits is considered an important issue for the development of the emu farming not only in Japan but also worldwide.

However, global reports reflect the scarcity of knowledge regarding the genetic improvement of the emu, which is attributable to relative recent domestication of emus compared to other primary livestock species. Despite some reports demonstrating the carcass traits of emu [6–8], large-scale data involving more than 300 slaughtered individuals are unavailable, except for those presented in our previous report [9]. Moreover, the reproductive characteristics and behavior of the emus may complicate genetic improvement programs. In addition to monogamy, emus exhibit polyandry, and males incubate the eggs [1,10], making accurate pedigree recording difficult for selective breeding. Furthermore, the rates of breeding success and egg laying are influenced by the compatibility between males and females [11]. On Japanese farms, large-scale rearing is generally conducted for effective reproduction, and eggs are collected and hatched using an incubator, making it difficult to record pedigree information for selective breeding. To resolve this issue, we previously developed a robust method for parentage tests in emu based on microsatellite markers [12]. Progeny testing is highly accurate method because it reflects the combined genetic influence on quantitative traits; however, it requires precise pedigree information. Given the diverse mating patterns above, applying progeny testing to emus can be time-consuming and expensive. In contrast, molecular marker-based approaches are effective only when the markers are robust and consistently applicable across different environments and management systems. Therefore, the development of DNA markers associated with the economic traits of emus is crucial for efficient genetic improvement.

The stearoyl-CoA desaturase (SCD) is a key enzyme regulating lipid metabolism, which catalyzes delta 9-desaturation of saturated fatty acids to monounsaturated fatty acids (MUFAs) [13]. Polymorphisms of the SCD encoding gene (SCD) are associated with fatty acid composition in cattle [14], pig [15,16], sheep [17], and chicken [18]. These reports suggest that *SCD* polymorphism is a potential DNA marker for selective breeding focused on fat production traits. However, no studies have documented *SCD* polymorphisms and their correlations with production traits in emus.

This study identified a non-synonymous substitution within exon 2 of the *SCD* gene in the emus and investigated the relationship between its genotypes and fat productivity. The c.267A > C substitution in the *SCD*, which resulted in an amino acid change, did not have a strong effect on fat production traits in our study population. Meanwhile, this study found that the *SCD* expression levels are associated with fat and meat production traits in emus.

## Materials and methods

### Ethics statement

All procedures involving animals met the guidelines described in “The Proper Conduct of Animal Experiments,” proposed by the Science Council of Japan and were approved by the Ethical Care and Use of Animals Committee at the Tokyo University of Agriculture (approval number: 270049, 280002, 290096, 300126, and 2019109). The study was carried out in compliance with the ARRIVE guidelines.

### Experimental birds

This study was conducted by using a part of data for body weight, fat yield, meat yield, and fat melting point data acquired from our previous study at Okhotsk Emu Farm, Abashiri, Hokkaido, Japan [9]. The emus used in this study were reared under similar management conditions, including diet and housing, as described in Koshiishi et al. [9]. These individuals were slaughtered between 2015 and 2017 at Hutami food factory, Abashiri, Hokkaido, Japan. Fat yield data were obtained from 266 (148 males and 118 females) emus slaughtered at two years of age. We used data on the melting points and fatty acid composition of the adipose tissue acquired from 164 and 25 individuals, respectively [9]. The fat melting point was measured according to our previous study that slip point method described by the National Livestock Breeding Center, Nishigo-mura, Fukushima, Japan (http://www.nlbc.go.jp/) [9]. Fatty acid composition of subcutaneous fat was investigated by gas chromatography-mass spectrometry (Agilent J&W DB-23 Columns, 7890A GC System, Agilent, USA), according to the conditions of previously reported methods [9,19]. Fatty acid composition analysis was performed on a subset of individuals (n = 25, 19 males and 6 females) due to limitations in sample availability and analytical cost. This analysis required several grams of adipose tissue per individual, unavailable for all samples. To reduce selection bias, individuals were selected to represent fat melting point ranges (low, intermediate, and high) [9]. Fatty acid composition was analyzed using combined datasets for males and females due to the limited sample size. Birds used for analysis were bred separately according to their age until they were slaughtered and kept in pens with free-access to the outdoor environment, and feed and water were provided *ad libitum*; the chemical components of the feed were described by Koshiishi *et al*. [9]. The birds were fasted for 10–12 h before slaughter. An operator measured the slaughtered body weight, subcutaneous and abdominal fat weight, and thigh meat production.

### Partial cDNA sequencing of *SCD*

The total RNA was extracted from the fat tissues collected from ten slaughtered individuals using the QIAzol reagent (Qiagen, Hilden, Germany) following the protocol provided by the manufacturer. After removing genomic DNA by DNase I treatment (Takara Bio, Ohtsu, Shiga, Japan) in total RNA, cDNAs were synthesized by using SuperScript VILO (Thermo Fisher Scientific, Waltham, MA, USA). RT-PCR was conducted in a 50 μL reaction mixture, comprising 1 × PCR buffer, 0.25 mM dNTPs, and 1 U PrimeTaq DNA polymerase (M & S Techno Systems, Osaka, Japan) with primers designed based on cDNA encoding emu SCD (XM_064513967) (S1 Table) under the following conditions: 95°C for 5 min, followed by 40 cycles of 94°C for 30 s, 55°C for 30 s and 72°C for 1.5 min, and a final extension step at 72°C for 5 min. PCR products were purified using a FastGene Gel/PCR Extraction Kit (NIPPON Genetics, Tokyo, Japan), followed by the determination of cDNA sequences using an ABI3730XL DNA Analyzer (Thermo Fisher Scientific, Waltham, MA, USA). To determine the 3’ terminal sequence of *SCD* that was not covered by RT-PCR, we designed oligonucleotide primers to amplify exon 5 based on genomic DNA sequence (ENSDNVT00000006468.1). Genomic DNA was extracted from ten slaughtered individuals using Isogenome (Nippon Gene, Tokyo, Japan). PCR was conducted in a 20 μL reaction mixture, comprising genomic DNA (0.05–10 ng), 1 × PCR buffer, 0.25 mM dNTPs, and 1 U PrimeTaq DNA polymerase (M & S Techno Systems), and primers listed in S1 Table, under the following conditions: 95°C for 5 min, followed by 40 cycles of 94°C for 30 s, 60°C for 30 s and 72°C for 30s, and a final extension step at 72°C for 5 min. PCR products were purified using ExoSAP IT (Thermo Fisher Scientific) and analyzed using a 3730XL DNA Analyzer (Thermo Fisher Scientific).

### Genotyping of *SCD*

Genomic DNA was extracted from the liver tissue of the slaughtered individuals using Isogenome (Nippon Gene). PCR was conducted in a 20 μL reaction mixture, comprising genomic DNA (0.05–10 ng), 1 × PCR buffer, 0.25 mM dNTPs, and 1 U PrimeTaq DNA polymerase (M & S Techno Systems), and primers listed in S1 Table, under the following conditions: 95°C for 5 min, followed by 40 cycles of 94°C for 30 s, 58°C for 30 s and 72°C for 1 min, and a final extension step at 72°C for 5 min. PCR primers were designed for the polymorphic site detected based on the prediction of the splice site through alignment of emu cDNA (XM_026094931.1) and chicken genomic DNA sequences (ENSGALG00000005739) for the *SCD* gene. PCR products were purified using ExoSAP IT (Thermo Fisher Scientific) and analyzed using a 3730XL DNA Analyzer (Thermo Fisher Scientific).

### Quantitative RT-PCR

Total RNA samples were extracted from fat tissues acquired from 46 of individuals, as described in the “Partial cDNA Sequencing of *SCD* section”. Quantitative RT-PCR (qRT-PCR) was conducted by using GeneAce SYBR™ qPCR Mix α Low ROX (Nippon Gene) and primers listed in S1 Table. PCR steps included 95°C for 10 min, followed by 45 cycles of 95°C for 30 s, 60°C for 1 min. The signal values were normalized to beta-actin (*ACTB*) and transformed using the ddCt method.

### Statistical analysis

Production traits were analyzed separately for males and females and as a combined dataset. In contrast, fatty acid composition was analyzed using combined datasets due to limited sample size. Sex differences in fat yield were analyzed using Welch’s *t*-test. Associations between production traits and *SCD* genotypes (AA, AC, and CC) were analyzed using one-way analysis of variance (ANOVA) followed by Tukey’s multiple comparison test. Correlations between production traits and *SCD* expression levels were evaluated using Pearson’s correlation coefficient. All statistical analyses were performed using the GraphPad Prism 9.5.1 (GraphPad Software, CA, USA).

## Results

### Identification of nucleotide substitution sites in *SCD*

**Fig 1:**
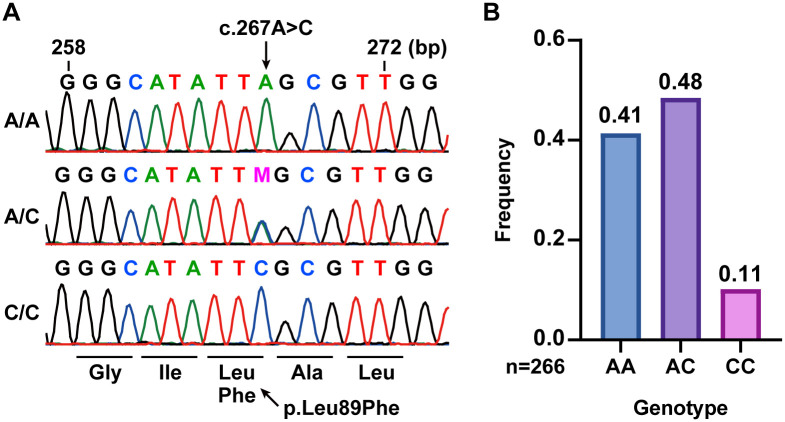
Polymorphism of the *SCD* gene in the emu. **(A)** Electropherograms of partial nucleotide sequences of *SCD* containing a polymorphic site in each genotype. Arrows indicated **c.**267A > C and p.Leu89Phe. **(B)** Genotype frequency of *SCD* detected in a Japanese farm population. Blue, purple, and pink bars showed genotype AA, AC, and CC, respectively.

We detected a 981 bp of partial DNA sequences of the *SCD* gene, which had a total CDS length of 1083-bp, and identified four nucleotide substitution sites in 10 individuals (XM_064513967; c.267A > C, c.576C > A, c.612C > T, and c.666G > A). Among them, c.267A > C was predicted to be a nonsynonymous substitution at the 89th codon, with leucine (Leu) replaced by phenylalanine (Phe) in the SCD protein (p.Leu89Phe) (Fig 1). Although the p.Leu89Phe substitution in the SCD protein was predicted to be functionally tolerated according to SIFT (https://sift.bii.a-star.edu.sg/) and PolyPhen-2 (http://genetics.bwh.harvard.edu/pph2/), we hypothesized that it is associated with fat production traits in emus. Next, we investigated the c.267A > C genotype in 266 emus using DNA sequencing. The genotype frequencies of AA, AC, and CC were 0.41, 0.48, and 0.11, respectively (Fig 1B). Despite the relatively higher frequency of AC heterozygosity in the study population, a few individuals exhibited the homozygous *C* allele. The genotype frequencies for the c.267A > C polymorphism in the *SCD* did not significantly deviate from Hardy–Weinberg equilibrium (χ² = 0.898, *P* = 0.343).

### Relationships between c.267A > C of *SCD* and carcass traits

**Fig 2:**
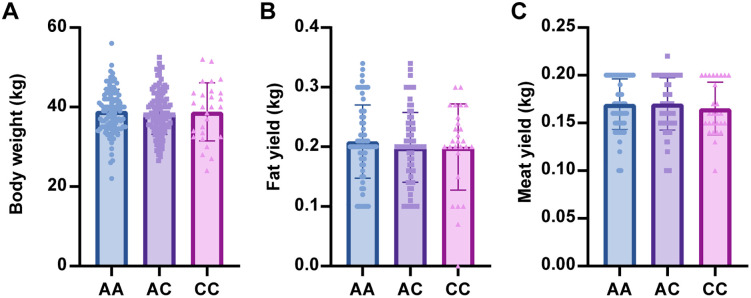
Relationships between *SCD* genotype and production traits in the emu. Body weight **(A)**, fat yield **(B)**, and meat yield **(C)** in each genotype. Blue, purple, and pink bars and dots show values from genotype AA, AC, and CC, respectively. No significant differences were observed in each genotype.

We investigated the association of the c.267A > C single-nucleotide polymorphism (SNP) with phenotypes by comparing body weight, fat yields (fat weight per 1 kg body weight), and meat yields (leg meat weight per 1 kg body weight) among the genotypes. The mean body weights±standard deviation (SD) were 38.99 ± 5.48, 38.53 ± 5.41, and 38.78 ± 7.31 kg in AA, AC, and CC genotypes, respectively, with no statistical differences detected among them (Fig 2A). The mean fat yields±SD of AA, AC, and CC were 0.21 ± 0.06, 0.20 ± 0.06, and 0.20 ± 0.06, respectively, with no significant differences among *SCD* genotypes (Fig 2B). The mean meat yields±SD (0.17 ± 0.03) did not significantly differ across the analyzed genotypes (Fig 2C).

**Fig 3:**
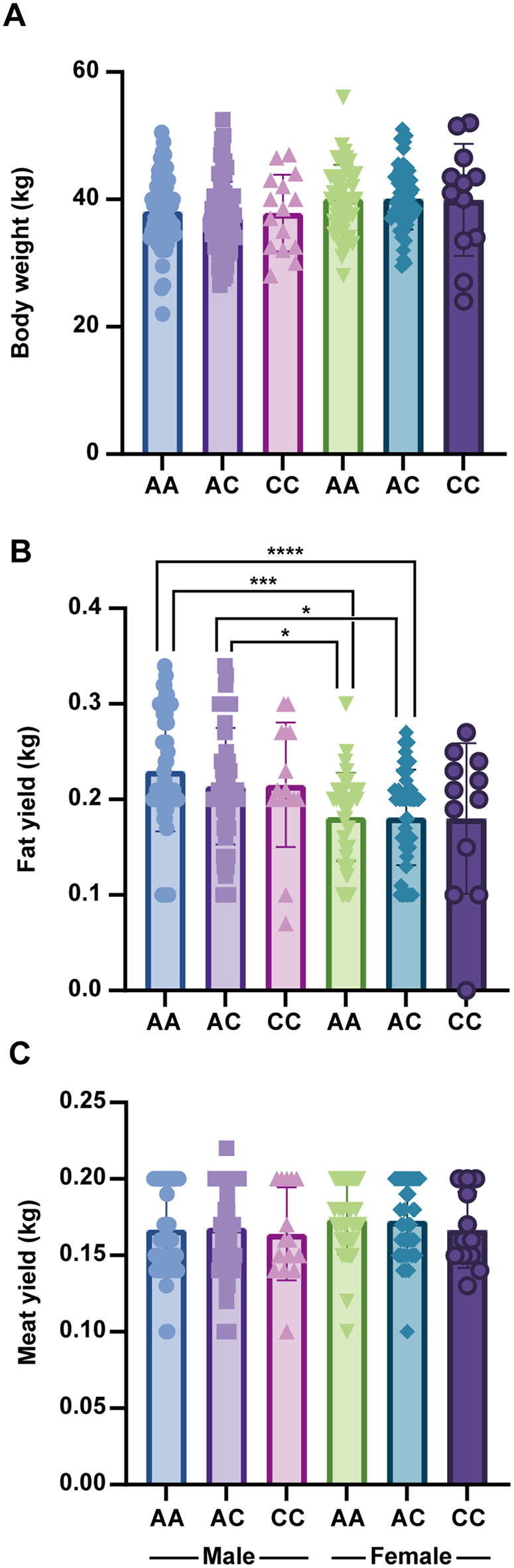
Relationships between *SCD* genotype and production traits in male and female emus. Body weight **(A)**, fat yield **(B)**, and meat yield **(C)** in each genotype. Blue, purple, pink, green, ice blue, and deep purple bars and dots indicated male AA, AC, CC, female AA, AC, and CC, respectively. *: P < 0.05, ***: P < 0.0001, ****: P < 0.00001. No significant differences were observed in each genotype.

As fat production is higher in male emus than in female ones [9], we estimated the sex differences in fat yields in the study population. The average fat yields±SD of males and females were 0.22 ± 0.06 and 0.18 ± 0.05, respectively, reflecting a significantly higher value in males than in females (P < 0.0001). Therefore, we separately analyzed the data corresponding to each sex. The mean body weight ± SD for the AA, AC, and CC genotypes was 38.12 ± 5.48, 37.25 ± 5.54, and 37.87 ± 6.03 kg in males, and 40.11 ± 5.32, 40.10 ± 4.85, and 39.92 ± 8.81 kg in females, respectively, with no significant differences among genotypes or between sexes (Fig 3A). The mean fat yields ± SD of the AA, AC, and CC genotypes were 0.23 ± 0.06, 0.21 ± 0.06, and 0.22 ± 0.07 kg in males, and 0.18 ± 0.05, 0.18 ± 0.05, and 0.18 ± 0.08 kg in females, respectively (Fig 3B). Despite a tendency for higher fat yields in the males with the AA genotype than in their counterparts with AC and CC genotypes, no significant intra-sex differences were observed among genotypes. Significantly higher values were observed in males with AA/AC than in females; however, no significant differences between males and females were recorded for the CC genotype. The observed differences in fat yield may be more strongly influenced by sex than by genotype, as males produce larger amounts of fat than females [9]. The mean meat yields ± SD of the AA, AC, and CC genotypes were 0.17 ± 0.03, 0.17 ± 0.03, and 0.16 ± 0.03 kg in males, and 0.17 ± 0.02 kg in females for all genotypes, with no significant differences among genotypes (Fig 3C). These results suggest that the c.267A > C variant of *SCD* does not have a considerable effect on fat or meat production traits in the emus.

### Relationships between c.267A > C of *SCD* and fat quality

**Fig 4:**
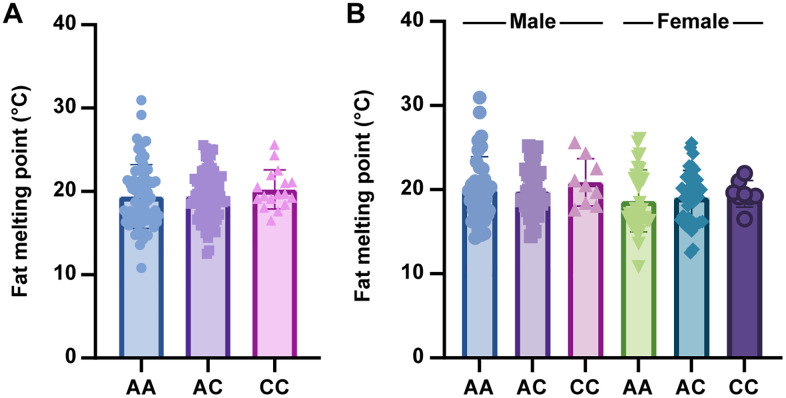
Relationships between *SCD* genotype and fat melting point. **(A)** Blue, purple, and pink bars and dots show values corresponding to the AA, AC, and CC genotypes, respectively. **(B)** Comparison of melting points among genotypes and genders. Blue, purple, pink, green, ice blue, and deep purple bars and dots indicated male AA, AC, CC, female AA, AC, and CC, respectively. No significant differences in fat melting point were observed across genotypes and genders.

SCD regulates the synthesis of unsaturated fatty acids [13], hence, we speculated that its polymorphism may be associated with fat melting temperature in the adipose tissue of the emu. The mean fat melting points±SD of individuals with AA, AC, and CC genotypes were 19.40 ± 3.83°C, 19.46 ± 2.91°C, and 20.22 ± 2.36°C, respectively (Fig 4A) with no significant differences among genotypes. Next, we investigated whether *SCD* genotypes affected the fat melting points in the data separated by sex. The mean fat melting points ± SD of the AA, AC, and CC genotypes were 20.01 ± 3.89, 19.77 ± 2.65, and 20.87 ± 2.81°C in males, and 18.65 ± 3.68, 19.08 ± 3.21, and 19.50 ± 1.59°C in females, respectively. No significant differences were identified across genotypes or sexes (Fig 4B).

**Fig 5:**
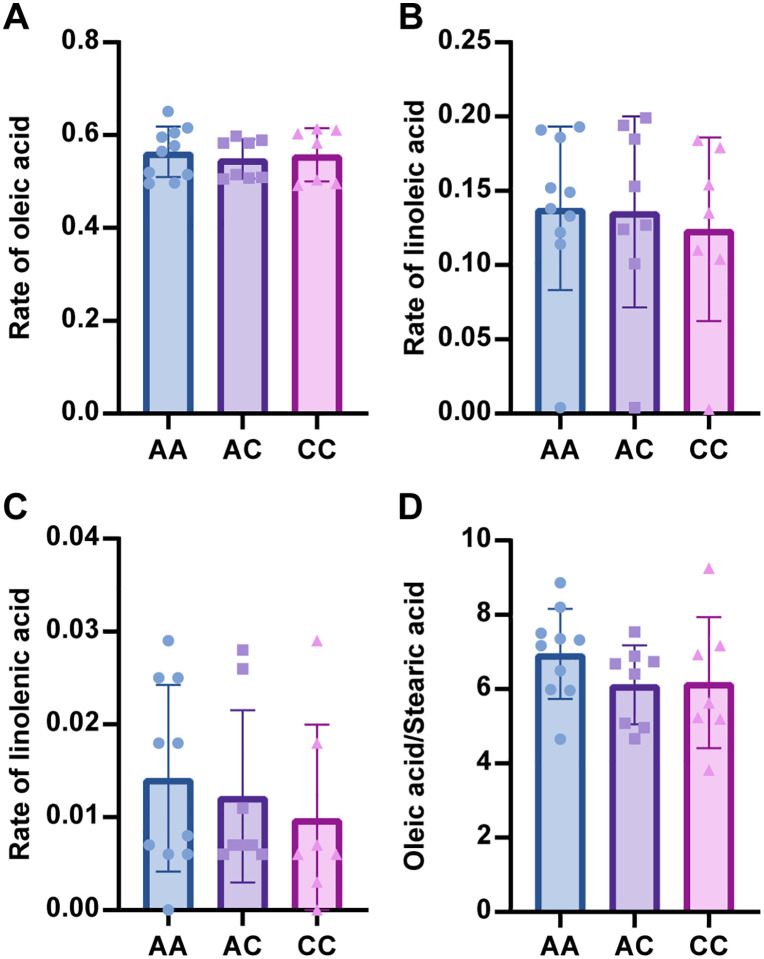
Relationships between *SCD* genotype and fatty acid composition. Relationship between the rates of oleic acid **(A)**, linoleic acid **(B)**, and linolenic acid **(C)** and *SCD* genotype. **(D)** Relationship between the ratio of oleic acid to stearic acid and *SCD* genotype. Blue, purple, and pink bars and dots show values corresponding to the AA, AC, and CC genotypes, respectively.

Furthermore, we investigated the association between the fatty acid composition and *SCD* genotypes. The mean rates of oleic acid±SD in individuals with AA, AC, and CC genotypes in *SCD* were 0.56 ± 0.05, 0.55 ± 0.04, and 0.56 ± 0.06, respectively (Fig 5A). The mean rates of linoleic acid±SD in individuals with AA, AC, and CC genotypes were 0.14 ± 0.06, 0.14 ± 0.06, and 0.12 ± 0.06, respectively (Fig 5B). The mean linolenic acid content±SD was similar among all *SCD* genotypes (0.01 ± 0.01; Fig 5C). No significant differences in any of the tested fatty acids were detected between the *SCD* genotypes. The ratios of oleic acid to stearic acid in the AA, AC, and CC genotypes were 6.95 ± 1.21, 6.12 ± 1.06, and 6.18 ± 1.76, respectively, with no significant differences (Fig 5D). These results suggest that the c.267A > C in *SCD* does not affect fatty acid composition in the emu.

### Relationships between expression levels of *SCD* and production traits

**Fig 6:**
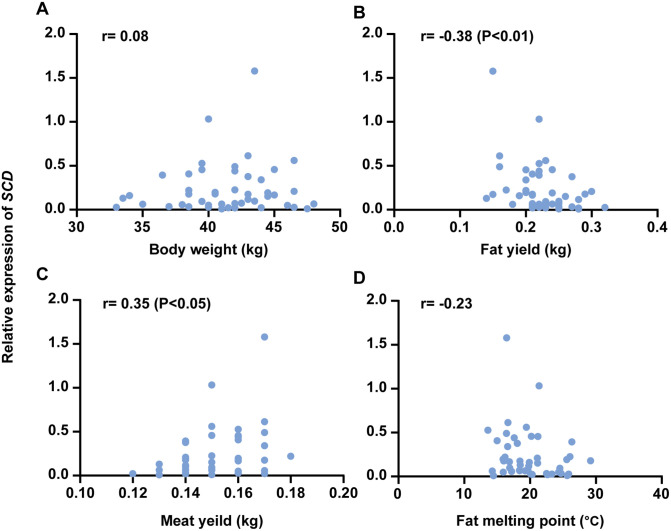
Relationships between production traits and *SCD* expression levels. Correlation between body weight **(A)**, fat yield **(B)**, meat yield **(C)**, and fat melting point **(D)** and *SCD* expression levels.

Next, we explored the correlation between the expression level of *SCD* and production traits, such as body weight, fat yield, meat yield, and fat melting point. No significant association was found between the c.267A > C genotypes and *SCD* expression levels, indicating no effect of c.267A > C on the expression levels of *SCD* transcript. Body weight was not correlated with *SCD* expression levels (Fig 6A). However, fat yield was negatively correlated with *SCD* expression levels (r = −0.38, P < 0.01) (Fig 6B). Conversely, *SCD* expression levels were positively correlated with meat yield (r = 0.35, P < 0.05) (Fig 6C). Although *SCD* expression levels tended to be negatively correlated with fat melting point, no significant association was detected (Fig 6D). These results suggest that SCD expression contributes to reduced fat deposition and increased meat yield in emus.

## Discussion

In this study, our results suggest that the *SCD* allele (c.267A > C) is not significantly associated with production traits in emus. Previous studies in poultry have reported that *SCD* polymorphisms are associated with fatty acid composition in intramuscular fat in Indonesian broiler chickens [20] and affect fatty acid content in thigh and breast meat in Korean native chickens [18]. These findings indicate that *SCD* plays an important role in regulating unsaturated fatty acid composition in avian species. However, to the best of our knowledge, no studies have elucidated the association between *SCD* polymorphisms and subcutaneous fat in birds. Intramuscular and abdominal fat depositions are regulated by different pathways in the chicken [21,22]. Luo et al. [21] suggested that abdominal fat deposition is associated with acetyl-CoA and glycerol metabolism-related pathways, which include SCD. However, the relatively small sample size may have limited statistical power to detect significant differences between genotypes. Fatty acid composition was investigated in pooled samples due to limited sample size. Although linolenic acid content showed a sex difference, no association between *SCD* genotype and linolenic acid content was observed in males. The relatively large standard deviations observed in some production traits may reflect individual variability and environmental influences. Production traits are complex quantitative traits influenced by multiple genes and environmental factors. While *SCD* may contribute to these traits, it is unlikely to act as the sole determinant. Other genes involved in lipid metabolism and their interactions may also play important roles, and further studies involving larger sample sizes and additional candidate genes are required to understand the genetic architecture underlying these traits in emus.

Meanwhile, we observed a significant negative correlation between fat yield and *SCD* expression levels (Fig 6). Expression levels of *SCD* transcripts increase during preadipocyte differentiation [23], and decreased *SCD* expression in cultured chicken preadipocytes results in reduced unsaturated fatty acid synthesis [24]. However, the mechanisms underlying the observed negative correlation between *SCD* expression levels and fat yield remain unclear. Environmental factors, including seasonal changes, may influence lipid metabolism in birds. In Northern Japan, air temperatures decline from October to November. The adipose tissues analyzed in this study were obtained from individuals slaughtered between September and November, a period during which adaptation to decreasing ambient temperatures and physiological changes associated with the onset of the breeding season are likely to occur simultaneously. However, the involvement of SCD in these processes remains unclear and requires further investigation. Further studies involving larger sample sizes and individuals from different farms are needed to validate these findings.

## Conclusion

In conclusion, we identified four SNPs and a non-synonymous substitution (c.267A > C; p.Leu89Phe) in the emu *SCD* gene. We reported that the *SCD* polymorphism (c.267A > C) is not significantly associated with production traits in emus. The expression levels of *SCD* are negatively and positively correlated with fat and meat yield, respectively. These findings may provide valuable insights into the genetic improvement of production traits in emus.

## Supporting information

S1 Table Primers for RT-PCR and cDNA sequence (A), genomic DNA sequence of exon 5 (B), c.267A > C genotyping (C), and qRT-PCR (D) in *SCD.* (XLSX)

S1 Data Raw datasets used in this study. The ZIP archive contains Data_01, Data_02, and Data_03.
(ZIP)
