# Supplementary material for: A polymorphism and expression levels of SCD and their relationships to production traits in a Japanese farmed emu population
Source: PLoS One. 2026 Jun 25;21(6):e0352695. doi: 10.1371/journal.pone.0352695 (PMC13298733; doi:10.1371/journal.pone.0352695)
Supplement: Attachment — Submitted filename: PONE-D-26-06317_reviewer.pdf [file pone.0352695.s004.pdf]

PLOS One

A polymorphism and expression levels of SCD and their relationships to production traits in a Japanese farmed emu population

--Manuscript Draft--

**Manuscript Number:**	PONE-D-26-06317
**Article Type:**	Research Article
**Full Title:**	A polymorphism and expression levels of SCD and their relationships to production traits in a Japanese farmed emu population
**Short Title:**	Relationships between SCD and production traits in emu
**Corresponding Author:**	Kenta Wada<br>Tokyo Nogyo Daigaku<br>Abashiri, JAPAN
**Keywords:**	emu, fat, fatty acids, polymorphism, stearoyl-CoA desaturase (SCD)
**Abstract:**	The emu is predicted to become a novel poultry species producing meat, eggs, and fat. In particular, emu oil derived from the fat is considered the most important product of the emu owing to its anti-inflammatory and anti-melanin production. Meanwhile, there are few reports on the genetic improvement of economic traits in emus. In this study, we discovered a non-synonymous substitution (c.267A>C, p.Leu89Phe) in the gene encoding stearoyl-CoA desaturase (SCD), a key enzyme involved in unsaturated fatty acid synthesis, in a Japanese farmed emu population. Although fat yields (fat weight per body weight) in males with AA and AC genotypes of SCD were higher than those in their female counterparts, significant intra-sex variations were not detected across genotypes in our study population. Moreover, fat melting points and fatty acid composition did not significantly vary between SCD genotypes. These results suggest that the SCD c.267A>C polymorphism does not affect fat production traits in emus. However, the expression levels of SCD transcripts were negatively correlated with fat content ($P < 0.01$) and positively correlated with meat yield ($P<0.05$), suggesting that increased SCD transcript levels in subcutaneous fat are associated with reduced fat content and higher meat production in emus.
**Order of Authors:**	Yuichi Koshiishi
	Suzuka Moriya
	Yuki Ishida
	Hiromitsu Ochiai
	Kenta Wada
**Additional Information:**	
**Question**	**Response**
**Financial Disclosure**<br><br>Enter a financial disclosure statement that describes the sources of funding for the work included in this submission. Review the submission guidelines for detailed requirements. View published research articles from *PLOS ONE* for specific examples.<br><br>This statement is required for submission and **will appear in the published article** if the submission is accepted. Please make	The author(s) received no specific funding for this work.

sure it is accurate.<br><br>**Funded studies**<br>Enter a statement with the following details:<br>• Initials of the authors who received each award<br>• Grant numbers awarded to each author<br>• The full name of each funder<br>• URL of each funder website<br>• Did the sponsors or funders play any role in the study design, data collection and analysis, decision to publish, or preparation of the manuscript?<br><br>Did you receive funding for this work?	
**Competing Interests**<br><br>Use the instructions below to enter a competing interest statement for this submission. On behalf of all authors, disclose any competing interests that could be perceived to bias this work—acknowledging all financial support and any other relevant financial or non-financial competing interests.<br><br>This statement is required for submission and **will appear in the published article** if the submission is accepted. Please make sure it is accurate and that any funding sources listed in your Funding Information later in the submission form are also declared in your Financial Disclosure statement.<br><br>View published research articles from *PLOS ONE* for specific examples.	The authors declare no conflict of interest.

Powered by Editorial Manager® and ProduXion Manager® from Aries Systems Corporation

**NO authors have competing interests**<br><br>Enter: *The authors have declared that no competing interests exist.*<br><br>**Authors with competing interests**<br><br>Enter competing interest details beginning with this statement:<br><br>*I have read the journal's policy and the authors of this manuscript have the following competing interests: [insert competing interests here]*<br><br>* typeset	
**Ethics Statement**<br><br>Enter an ethics statement for this submission. This statement is required if the study involved:<br><br>• Human participants<br>• Human specimens or tissue<br>• Vertebrate animals or cephalopods<br>• Vertebrate embryos or tissues<br>• Field research<br><br>Write "N/A" if the submission does not require an ethics statement.<br><br>General guidance is provided below. Consult the submission guidelines for detailed instructions. **Make sure that all information entered here is included in the Methods section of the manuscript.**	Ethics statement<br>All procedures involving animals met the guidelines described in "The Proper Conduct of Animal Experiments," proposed by the Science Council of Japan and were approved by the Ethical Care and Use of Animals Committee at the Tokyo University of Agriculture (approval number: 270049, 280002, 290096, 300126, and 2019109). The study was carried out in compliance with the ARRIVE guidelines.

Powered by Editorial Manager® and ProduXion Manager® from Aries Systems Corporation

**Format for specific study types**<br><br>**Human Subject Research (involving human participants and/or tissue)**<br>• Give the name of the institutional review board or ethics committee that approved the study<br>• Include the approval number and/or a statement indicating approval of this research<br>• Indicate the form of consent obtained (written/oral) or the reason that consent was not obtained (e.g. the data were analyzed anonymously)<br><br>**Animal Research (involving vertebrate animals, embryos or tissues)**<br>• Provide the name of the Institutional Animal Care and Use Committee (IACUC) or other relevant ethics board that reviewed the study protocol, and indicate whether they approved this research or granted a formal waiver of ethical approval<br>• Include an approval number if one was obtained<br>• If the study involved *non-human primates*, add *additional details* about animal welfare and steps taken to ameliorate suffering<br>• If anesthesia, euthanasia, or any kind of animal sacrifice is part of the study, include briefly which substances and/or methods were applied<br><br>**Field Research**<br><br>Include the following details if this study involves the collection of plant, animal, or other materials from a natural setting:<br>• Field permit number<br>• Name of the institution or relevant body that granted permission	
**Data Availability**<br><br>Authors are required to make all data underlying the findings described fully available, without restriction, and from the time of publication. PLOS allows rare exceptions to address legal and ethical concerns. See the PLOS Data Policy and FAQ for detailed information.	Yes - all data are fully available without restriction

A Data Availability Statement describing where the data can be found is required at submission. Your answers to this question constitute the Data Availability Statement and **will be published in the article**, if accepted.<br><br>**Important:** Stating 'data available on request from the author' is not sufficient. If your data are only available upon request, select 'No' for the first question and explain your exceptional situation in the text box.<br><br>Do the authors confirm that all data underlying the findings described in their manuscript are fully available without restriction?	
**Describe where the data may be found in full sentences. If you are copying our sample text, replace any instances of XXX with the appropriate details.**<br><br>• If the data are **held or will be held in a public repository**, include URLs, accession numbers or DOIs. If this information will only be available after acceptance, indicate this by ticking the box below. For example: *All XXX files are available from the XXX database (accession number(s) XXX, XXX.).*<br>• If the data are all contained **within the manuscript and/or Supporting Information files**, enter the following: *All relevant data are within the manuscript and its Supporting Information files.*<br>• If neither of these applies but you are able to provide **details of access elsewhere**, with or without limitations, please do so. For example:<br><br>*Data cannot be shared publicly because of [XXX]. Data are available from the XXX Institutional Data Access / Ethics Committee (contact via XXX) for researchers who meet the criteria for access to confidential data.*<br><br>*The data underlying the results presented in the study are available from (include the name of the third party*	All relevant data are within the manuscript and its Supporting Information files.

and contact information or URL).<br>• This text is appropriate if the data are owned by a third party and authors do not have permission to share the data.<br><br>* typeset	
Additional data availability information:	

Powered by Editorial Manager® and ProduXion Manager® from Aries Systems Corporation

Manuscript

Click here to access/download;Manuscript;Koshiishi_et_al_Manuscript.docx

A polymorphism and expression levels of *SCD* and their relationships to production traits in a Japanese farmed emu population

Yuichi Koshiishi[1], Suzuka Moriya[2#], Yuki Ishida[3], Hiromitsu Ochiai[3], and Kenta Wada[2, 3*]

1 International Institute for Zoonosis Control, Hokkaido University, Sapporo, Hokkaido, Japan

2 Faculty of Bioindustry, Tokyo University of Agriculture, Abashiri, Hokkaido, Japan,

3 Graduate School of Bioindustry, Tokyo University of Agriculture, Abashiri, Hokkaido, Japan

* Corresponding author

E-mail: k3wada@nodai.ac.jp (KW)

#Current affiliation: Division of Applied Biosciences, Graduate School of Agriculture, Kyoto University, Sakyo-ku, Kyoto, Japan

Short title: Relationships between *SCD* and production traits in emu.

Abstract

The emu is predicted to become a novel poultry species producing meat, eggs, and fat. In particular, emu oil derived from the fat is considered the most important product of the emu owing to its anti-inflammatory and anti-melanin production. Meanwhile, there are few reports on the genetic improvement of economic traits in emus. In this study, we discovered a non-synonymous substitution (c.267A>C, p.Leu89Phe) in the gene encoding stearoyl-CoA desaturase (SCD), a key enzyme involved in unsaturated fatty acid synthesis, in a Japanese farmed emu population. Although fat yields (fat weight per body weight) in males with AA and AC genotypes of *SCD* were higher than those in their female counterparts, significant intra-sex variations were not detected across genotypes in our study population. Moreover, fat melting points and fatty acid composition did not significantly vary between *SCD* genotypes. These results suggest that the *SCD* c.267A>C polymorphism does not affect fat production traits in emus. However, the expression levels of *SCD* transcripts were negatively correlated with fat content ($P < 0.01$) and positively correlated with meat yield ($P<0.05$), suggesting that

increased *SCD* transcript levels in subcutaneous fat are associated with reduced fat content and higher meat production in emus.

Introduction

The emu (*Dromaius novaehollandiae*), which originated in Australia, is the second-largest bird species and belongs to the ratite family. In the 1970s, Australia made an initial attempt to utilize emus for leather production. Subsequently, the industrial application for meat, egg, and oil production using emus expanded to other countries, such as the USA, Europe, Asia, and Africa [1]. Among their products, oil purified from adipose tissue is highly valued as a raw material for cosmetic and skincare products because it has been reported to exhibit anti-inflammatory [2–4] and anti-melanogenic effects [5]. Therefore, the genetic improvement of oil production-related traits is considered an important issue for development of the emu farming not only in Japan but also worldwide.

However, global reports reflect the scarcity of knowledge regarding the genetic improvement of the emu, which is attributable to relative recent domestication of emus compared to other primary livestock species. Despite some reports demonstrating the carcass traits of emu [6–8], large-scale data involving more than 300 slaughtered individuals are unavailable, except for those presented in our previous report [9].

Moreover, the reproductive characteristics and behavior of the emus may have potentially limited the elucidation of their genetic improvement. In addition to monogamy, emus exhibit polyandry, and the laid eggs are incubated by males [1,10]. Furthermore, the rates of breeding success and egg laying are influenced by the compatibility ~~(chemistry)~~ between males and females [11]. On Japanese farms, large-scale rearing is generally conducted for effective reproduction, and eggs are collected and hatched using incubator, making it difficult to record pedigree information for selective breeding. To resolve this issue, we previously developed a robust method for parentage tests in emu based on microsatellite markers ~~in the emu~~ [12]. However, progeny test-based selective breeding is a time-consuming method for improving production traits. Hence, the development of DNA markers associated with the economic traits of emus is crucial for efficient genetic improvement.

The stearoyl-CoA desaturase (SCD) is a key enzyme regulating lipid metabolism, which catalyzes delta 9-desaturation of saturated fatty acids to monounsaturated fatty acids (MUFAs) [13]. Polymorphisms of the SCD encoding gene (*SCD*) are associated with fatty acid composition in cattle [14], pig [15,16], sheep [17],

and chicken [18]. These reports suggest that *SCD* polymorphism is a potential DNA marker for selective breeding focused on fat production traits. However, no studies have documented *SCD* polymorphisms and their correlations with production traits in emus.

This study identified a non-synonymous substitution within exon 2 of the *SCD* gene in the emus and investigated the relationship between its genotypes and fat productivity. The c.267A>C substitution in the *SCD*, which resulted in an amino acid change, did not have a strong effect on fat production traits in our study population. Meanwhile, this study found that the *SCD* expression levels are associated with fat and meat production traits in emus.

Materials and methods

Experimental birds

This study was conducted by using a part of data for body weight, fat yield, meat yield, and fat melting point data acquired from our previous study at Okhotsk Emu Farm, Abashiri, Hokkaido, Japan [9]. Fat yield data were obtained from 266 emus slaughtered at two years of age in 2015 and 2017. We used data on the melting points and fatty acid composition of the adipose tissue acquired from 168 and 25 individuals, respectively[9]. Birds used for analysis were bred separately according to their age until they were slaughtered and kept in pens with free-access to the outdoor environment, and feed and water were provided *ad libitum*; the chemical components of the feed were described by Koshiishi *et al*. ~~(2022)~~ [9]. The birds were fasted for 10–12 h before slaughter.

Partial cDNA sequencing of *SCD*

The total RNA was extracted from the fat tissues collected from ten slaughtered individuals using the QIAzol reagent (Qiagen, Hilden, Germany) following the

protocol provided by the manufacturer. After removing genomic DNA by DNase I treatment (Takara Bio, Ohtsu, Shiga, Japan) in total RNA, cDNAs were synthesized by using SuperScript VILO (Thermo Fisher Scientific, Waltham, MA, USA). RT-PCR was conducted in a 50 μL reaction mixture, comprising 1 × PCR buffer, 0.25 mM dNTPs, and 1 U PrimeTaq DNA polymerase (M & S Techno Systems, Osaka, Japan) with primers designed based on cDNA encoding emu SCD (XM_064513967) (S1 Table) under the following conditions: 95°C for 5 min, followed by 40 cycles of 94°C for 30 s, 55°C for 30 s and 72°C for 1.5 min, and a final extension step at 72°C for 5 min. PCR products were purified using a FastGene Gel/PCR Extraction Kit (NIPPON Genetics, Tokyo, Japan), followed by the determination of cDNA sequences using an ABI3730XL DNA Analyzer (Thermo Fisher Scientific, Waltham, MA, USA). To determine the 3’ terminal sequence of *SCD* that was not covered by RT-PCR, we designed oligonucleotide primers to amplify exon 5 based on genomic DNA sequence (ENSDNVT00000006468.1). Genomic DNA was extracted from ten slaughtered individuals using Isogenome (Nippon Gene, Tokyo, Japan). PCR was conducted in a 20 μL reaction mixture, comprising genomic DNA (0.05–10 ng), 1 × PCR buffer, 0.25 mM

dNTPs, and 1 U PrimeTaq DNA polymerase (M & S Techno Systems), and primers listed in S1 Table, under the following conditions: 95°C for 5 min, followed by 40 cycles of 94°C for 30 s, 60°C for 30 s and 72°C for 30s, and a final extension step at 72°C for 5 min. PCR products were purified using ExoSAP IT (Thermo Fisher Scientific) and analyzed using a 3730XL DNA Analyzer (Thermo Fisher Scientific).

Genotyping of *SCD*

Genomic DNA was extracted from the liver tissue of the slaughtered individuals using Isogenome (Nippon Gene). PCR was conducted in a 20 μL reaction mixture, comprising genomic DNA (0.05–10 ng), 1 × PCR buffer, 0.25 mM dNTPs, and 1 U PrimeTaq DNA polymerase (M & S Techno Systems), and primers listed in S1 Table, under the following conditions: 95°C for 5 min, followed by 40 cycles of 94°C for 30 s, 58°C for 30 s and 72°C for 1 min, and a final extension step at 72°C for 5 min. PCR primers were designed for the polymorphic site detected based on the prediction of the splice site through alignment of emu cDNA (XM_026094931.1) and chicken genomic DNA

sequences (ENSGALG00000005739) for the *SCD* gene. PCR products were purified using ExoSAP IT (Thermo Fisher Scientific) and analyzed using a 3730XL DNA Analyzer (Thermo Fisher Scientific).

Quantitative RT-PCR

Total RNA samples were extracted from fat tissues acquired from 46 of individuals, as described in the “Partial cDNA Sequencing of *SCD* section”. Quantitative RT-PCR (qRT-PCR) was conducted by using GeneAce SYBR™ qPCR Mix α Low ROX (Nippon Gene) and primers listed in S1 Table. PCR steps included 95°C for 10 min, followed by 45 cycles of 95°C for 30 s, 60°C for 1 min. The signal values were normalized to beta-actin (*ACTB*) and transformed using the ddCt method.

Statistical analysis

The association between production traits and genotypes was analyzed using one-way Analysis of Variance (ANOVA) with Tukey's multiple comparison test. The sex difference in fat yields was confirmed using Welch’s *t*-test. All statistical analyses were performed using the GraphPad Prism 9.5.1 (GraphPad Software, CA, USA).

Results

Identification of nucleotide substitution sites in *SCD*

We detected a 981 bp of partial DNA sequences of the *SCD* gene, which had a total CDS length of 1083-bp, and identified four nucleotide substitution sites in 10 individuals (XM_064513967; c.267A>C, c.576C>A, c.612C>T, and c.666G>A). Among them, c.267A>C was predicted to be a nonsynonymous substitution at the 89th codon, with leucine (Leu) replaced by phenylalanine (Phe) in the SCD protein (p.Leu89Phe) (Fig 1A). Although the p.Leu89Phe substitution in the SCD protein was predicted to be functionally tolerated according to SIFT (https://sift.bii.a-star.edu.sg/) and PolyPhen-2 (http://genetics.bwh.harvard.edu/pph2/), we hypothesized that it is associated with fat production traits in emus. Next, we investigated the c.267A>C genotype in 266 emus using DNA sequencing. The genotype frequencies of AA, AC, and CC were 0.41, 0.48, and 0.11, respectively (Fig 1B). Despite the relatively higher frequency of AC heterozygosity in the study population, a few individuals exhibited the homozygous *C* allele.

Fig 1. Polymorphism of the *SCD* gene in the emu. (A) Electropherograms of partial nucleotide sequences of *SCD* containing a polymorphic site in each genotype. Arrows indicated c.267A>C and p.Leu89Phe. **(B)** Genotype frequency of *SCD* detected in a Japanese farm population. Blue, purple, and pink bars showed genotype AA, AC, and CC, respectively.

Relationships between c.267A>C of *SCD* and carcass traits

We investigated the association of the c.267A>C single-nucleotide polymorphism (SNP) with phenotypes by comparing body weight, fat yields (fat weight per 1 kg body weight), and meat yields (leg meat weight per 1 kg body weight) among the genotypes. The mean body weights±standard deviation (SD) were 38.99±5.48, 38.53±5.41, and 38.78±7.31 kg in AA, AC, and CC genotypes, respectively, with no statistical differences detected among them (Fig 2A). The mean fat yields±SD of AA, AC, and CC were 0.21±0.06, 0.20±0.06, and 0.20±0.06, respectively, with no significant differences

among *SCD* genotypes (Fig 2B). The mean meat yields±SD (0.17±0.03) did not significantly differ across the analyzed genotypes (Fig 2C).

Fig 2. Relationships between *SCD* genotype and production traits in the emu. Body weight (**A**), fat yield (**B**), and meat yield (**C**) in each genotype. Blue, purple, and pink bars and dots show values from genotype AA, AC, and CC, respectively. No significant differences were observed in each genotype.

As fat production is higher in male emus than in female ones [9], we estimated the sex differences in fat yields in the study population. The average fat yields±SD of males and females were 0.22±0.06 and 0.18±0.05, respectively, reflecting a significantly higher value in males than in females ($P<0.0001$). Therefore, we separately analyzed the data corresponding to each sex. The mean body weight ± SD for the AA, AC, and CC genotypes was 38.12 ± 5.48, 37.25 ± 5.54, and 37.87 ± 6.03 kg in males, and 40.11 ± 5.32, 40.10 ± 4.85, and 39.92 ± 8.81 kg in females, respectively, with no

significant differences among genotypes or between genders (Fig 3A). The mean fat yields ± SD of the AA, AC, and CC genotypes were 0.23 ± 0.06, 0.21 ± 0.06, and 0.22 ± 0.07 kg in males, and 0.18 ± 0.05, 0.18 ± 0.05, and 0.18 ± 0.08 kg in females, respectively (Fig 3B). Despite a tendency for higher fat yields in the males with the AA genotype than in their counterparts with AC and CC genotypes, no significant intra-sex differences were observed among genotypes. Significantly higher values were observed in males with AA/AC than in females; however, no significant differences between males and females were recorded for the CC genotype. The observed differences in fat yield may be more strongly influenced by sex than by genotype, as males produce larger amounts of fat than females [9]. The mean meat yields ± SD of the AA, AC, and CC genotypes were 0.17 ± 0.03, 0.17 ± 0.03, and 0.16 ± 0.03 kg in males, and 0.17 ± 0.02 kg in females for all genotypes, with no significant differences among genotypes (Fig 3C). These results suggest that the c.267A>C variant of *SCD* does not have a considerable effect on fat or meat production traits in the emus.

Fig 3. Relationships between *SCD* genotype and production traits in male and female emus. Body weight (**A**), fat yield (**B**), and meat yield (**C**) in each genotype. Blue, purple, pink, green, ice blue, and deep purple bars and dots indicated male AA, AC, CC, female AA, AC, and CC, respectively. *: $P<0.05$, ***: $P<0.0001$, ****: $P<0.00001$. No significant differences were observed in each genotype.

Relationships between c.267A>C of *SCD* and fat quality

SCD regulates the synthesis of unsaturated fatty acids [13], hence, we speculated that its polymorphism may be associated with fat melting temperature in the adipose tissue of the emu. The mean fat melting points±SD of individuals with AA, AC, and CC genotypes were 19.40±3.83℃, 19.46±2.91℃, and 20.22±2.36℃, respectively (Fig 4A). A tendency toward lower melting points in AA/AC groups was detected compared to the CC counterparts; however, no significant differences were observed. Next, we investigated whether *SCD* genotypes affected the fat melting points in the data separated by sex. The mean fat melting points ± SD of the AA, AC, and CC genotypes were 20.01

± 3.89, 19.77 ± 2.65, and 20.87 ± 2.81°C in males, and 18.65 ± 3.68, 19.08 ± 3.21, and 19.50 ± 1.59°C in females, respectively. Despite a tendency for lower fat melting point in females than in males, no significant differences were identified across genotypes or sexes (Fig 4B).

Fig 4. Relationships between *SCD* genotype and fat melting point. (A) Blue, purple, and pink bars and dots show values corresponding to the AA, AC, and CC genotypes, respectively. **(B)** Comparison of melting points among genotypes and genders. Blue, purple, pink, green, ice blue, and deep purple bars and dots indicated male AA, AC, CC, female AA, AC, and CC, respectively. No significant differences in fat melting point were observed across genotypes and genders.

Furthermore, we investigated the association between the fatty acid composition and *SCD* genotypes. The mean rates of oleic acid±SD in individuals with AA, AC, and CC genotypes in *SCD* were 0.56±0.05, 0.55±0.04, and 0.56±0.06,

respectively (Fig 5A). The mean rates of linoleic acid±SD in individuals with AA, AC, and CC genotypes were 0.14±0.06, 0.14±0.06, and 0.12±0.06, respectively (Fig 5B). The mean linolenic acid content±SD was similar among all *SCD* genotypes (0.01 ± 0.01; Fig 5C). No significant differences in any of the tested fatty acids were detected between the *SCD* genotypes. The ratios of oleic acid to stearic acid in the AA, AC, and CC genotypes were 6.95±1.21, 6.12±1.06, and 6.18±1.76, respectively, with no significant differences (Fig 5D). These results suggest that the c.267A>C in *SCD* does not affect fatty acid composition in the emu.

Fig 5. Relationships between *SCD* genotype and fatty acid composition.

Relationship between the rates of oleic acid (**A**), linoleic acid (**B**), and linolenic acid (**C**) and *SCD* genotype. (**D**) Relationship between the ratio of oleic acid to stearic acid and *SCD* genotype. Blue, purple, and pink bars and dots show values corresponding to the AA, AC, and CC genotypes, respectively.

Relationships between expression levels of *SCD* and production traits

Next, we explored the correlation between the expression level of *SCD* and production traits, such as body weight, fat yield, meat yield, and fat melting point. No significant association was found between the c.267A>C genotypes and *SCD* expression levels, indicating no effect of c.267A>C on the expression levels of *SCD* transcript. Body weight was not correlated with *SCD* expression levels (Fig 6A). However, fat yield was negatively correlated with *SCD* expression levels (r=-0.38, P<0.01) (Fig 6B). Conversely, *SCD* expression levels were positively correlated with meat yield (r=0.35, P<0.05) (Fig 6C). Although *SCD* expression levels tended to be negatively correlated with fat melting point, no significant association was detected (Fig 6D). These results suggest that SCD expression contributes to reduced fat deposition and increased meat yield in emus.

Fig. 6. Relationships between production traits and *SCD* expression levels.

Correlation between body weight (**A**), fat yield (**B**), meat yield (**C**), and fat melting point (**D**) and *SCD* expression levels.

Discussion

In this study, our results suggest that the *SCD* genotype (c.267A>C) is not associated with production traits in emus. The *SCD* polymorphism exhibits additive effects on fatty acid composition and melting temperature of intramuscular fat in the Japanese Black cattle [14]. In the pigs, *SCD* polymorphisms are associated with the fatty acid composition of subcutaneous adipose tissues, and the carcass weights [15,16]. These previous reports reflect the significant role of *SCD* in unsaturated fatty acid contents in both intramuscular and subcutaneous fat tissues in mammals. In the Korean native chickens, *SCD* polymorphisms affects the fatty acid content of the thigh and breast meat [18]. Intramuscular and abdominal fat deposition are regulated by different pathways in the chicken [19,20]. Luo et al. (2022) suggested that abdominal fat deposition is associated with acetyl-CoA and glycerol metabolism-related pathways, which include SCD. To the best of our knowledge, there have been no reports elucidated the association between *SCD* polymorphisms and subcutaneous fat in birds. Consequently, we concluded that the *SCD* polymorphism identified in this study was not associated with fat production traits in emus.

Meanwhile, we indicated that higher expression levels of *SCD* potentially contribute to reduced fat deposition and increased meat production (Fig 6). Expression levels of *SCD* transcripts increases during preadipocyte differentiation [21]. Pigs exhibit high expression levels of *SCD* transcripts in their adipose tissues [22]. Previous reports have demonstrated that high *SCD* expression is associated with increased adipogenesis. Although we could not explain the negative correlation between *SCD* expression levels and fat yield. In Northern Japan, air temperatures decline from October to November. Mammals reduce the proportion of saturated fatty acids and increase the proportion of unsaturated fatty acids in subcutaneous adipose tissue as an adaptation to cold environments [23]. Fatty acid desaturation lowers the melting point of fat and is considered important for maintaining adipose tissue fluidity under low-temperature conditions [23]. The adipose tissues analyzed in this study were obtained from individuals slaughtered between September and November, a period during which adaptation to decreasing ambient temperatures and physiological changes associated with the onset of the breeding season are likely to occur simultaneously. Therefore, the negative correlation observed between *SCD* expression levels and fat yield in this study

may reflect the concurrent occurrence of fatty acid desaturation for thermoregulation and reproduction-associated lipid metabolism (lipolysis with increased *SCD* expression). Indeed, although the correlation was not statistically significant, the expression levels of *SCD* transcripts tended to be associated with fat melting temperature (Fig 6D). As SCD catalyzes the conversion of saturated fatty acids into monounsaturated fatty acids [13], its expression levels may influence the subcutaneous fat melting temperature in the emus. In contrast, *SCD* expression levels were positively correlated with meat yield (Fig 6C). *SCD* polymorphisms are associated with carcass weight in heavy pigs [24]. Therefore, the expression level of *SCD* transcripts may be associated with meat production in emus.

Further studies involving larger sample sizes and using individuals from different farms may effectively validate our results. In addition, identifying the genetic factors that regulate *SCD* transcription is crucial for the development of markers for the selective breeding of emus.

Conclusion

In conclusion, we identified four SNPs and a non-synonymous substitution (c.267A>C; p.Leu89Phe) in the emu *SCD* gene. We suggest that the *SCD* polymorphism (c.267A>C) is not associated with production traits in emus. The expression levels of *SCD* are negatively and positively correlated with fat and meat yield, respectively. Despite the lack of statistical significance, fat melting temperature tended to be associated with *SCD* expression levels. These findings may provide valuable insights into the genetic improvement of production traits in emus.

Acknowledgements

We thank Okhotsk Emu Farm and Tokyo Nodai Startup Bioindustry Co., Ltd (Abashiri, Hokkaido, Japan) for their assistance with sample collection. We thank Yuki Nishiyama, Sunao Tamura, and Nozomi Kimura for their technical support. This work was supported by the Grant-in-Aid for the Abashiri Cold Region Farm Research Project and the Grant-in-Aid for University Strategic Study Project from Tokyo University of Agriculture. We would like to thank Editage (www.editage.com) for the English language editing.

References

1. Sales J. The emu (Dromaius novaehollandiae): a review of its biology and commercial products. Avian Poult Biol Rev. 2007;18: 1–20.

2. Snowden JM, Whitehouse MW. Anti-inflammatory activity of emu oils in rats. Inflammopharmacology. 1997;5: 127–132.

3. Abimosleh SM, Tran CD, Howarth GS. Emu Oil: a novel therapeutic for disorders of the gastrointestinal tract? J Gastroenterol Hepatol. 2012;27: 857–861.

4. Miyashita T, Minami K, Ito M, Koizumi R, Sagane Y, Watanabe T, et al. Emu Oil Reduces LPS-Induced Production of Nitric Oxide and TNF-α but not Phagocytosis in RAW 264 Macrophages. J Oleo Sci. 2018;67: 471–477.

5. Ito M, Minami K, Sagane Y, Watanabe T, Niwa K. Data on melanin production in B16F1 melanoma cells in the presence of emu oil. Data Brief. 2016;9: 1056–1059.

6. Naveena BM, Sen AR, Muthukumar M, Girish PS, Praveen Kumar Y, Kiran M. Carcass characteristics, composition, physico-chemical, microbial and sensory quality of emu meat. Br Poult Sci. 2013;54: 329–336.

7. Menon DG, Bennett DC, Uttaro B, Schaefer AL, Cheng KM. Carcass yields and meat quality characteristics of adult emus (Dromaius novaehollandiae) transported for 6h before slaughter. Meat Sci. 2014;98: 240–246.

8. Beckerbauer LM, Thiel-Cooper R, Ahn DU, Sell JL, Parrish FC Jr, Beitz DC. Influence of two dietary fats on the composition of emu oil and meat. Poult Sci. 2001;80: 187–194.

9. Koshiishi Y, Murata-Okubo M, Nakajyo H, Machida R, Yamamoto T, Myoda T, et al. Carcass traits and fat quality of breeding emu (Dromaius novaehollandiae) in Northern Japan. Poult Sci. 2022;101: 102050.

10. Taylor EL, Blache D, Groth D, Wetherall JD, Martin GB. Genetic evidence for mixed parentage in nests of the emu (Dromaius novaehollandiae). Behav Ecol Sociobiol. 2000;47: 359–364.

11. Yokohama M, Jinushi H, Imai S, Ikeya K. Effects of Pairing on Egg Laying in the Emu. Journal of Agriculture Science, Tokyo University of Agriculture. 2014;58: 229–234.

12. Koshiishi Y, Uchiyama H, Murata-Okubo M, Tanaka K, Kameyama Y, Hirayama H, et al. Development of 49 novel microsatellite markers from Next-generation sequencing data and a robust method for parentage tests in the emu (Dromaius novaehollandiae). Gene. 2021;769: 145238.

13. Ntambi JM. The role of Stearoyl-CoA desaturase in hepatic de novo lipogenesis. Biochem Biophys Res Commun. 2022;633: 81–83.

14. Mannen H. Identification and utilization of genes associated with beef qualities. Anim Sci J. 2011;82: 1–7.

15. Renaville B, Bacciu N, Lanzoni M, Mossa F, Piasentier E. Association of single nucleotide polymorphisms in fat metabolism candidate genes with fatty acid profiles of muscle and subcutaneous fat in heavy pigs. Meat Sci. 2018;139: 220–227.

16. Solé E, González-Prendes R, Oliinychenko Y, Tor M, Ros-Freixedes R, Estany J, et al. Transcriptome shifts triggered by vitamin A and SCD genotype interaction in Duroc pigs. BMC Genomics. 2022;23: 16.

17. Al-Thuwaini TM, Al-Shuhaib MBS. Variants of the SCD gene and their association with fatty acid composition in Awassi sheep. Mol Biol Rep. 2022;49: 7807–7813.

18. Jin S, Lee SH, Lee DH, Manjula P, Lee SH, Lee JH. Genetic association of DEGS1, ELOVL6, FABP3, FABP4, FASN and SCD genes with fatty acid composition in breast and thigh muscles of Korean native chicken. Anim Genet. 2020;51: 344–345.

19. Luo N, Shu J, Yuan X, Jin Y, Cui H, Zhao G, et al. Differential regulation of intramuscular fat and abdominal fat deposition in chickens. BMC Genomics. 2022;23: 308.

20. Zhang M, Li F, Ma X-F, Li W-T, Jiang R-R, Han R-L, et al. Identification of differentially expressed genes and pathways between intramuscular and abdominal fat-derived preadipocyte differentiation of chickens in vitro. BMC Genomics. 2019;20: 743.

21. Casimir DA, Ntambi JM. cAMP activates the expression of stearoyl-CoA desaturase gene 1 during early preadipocyte differentiation. J Biol Chem. 1996;271: 29847–29853.

22. Smith SB, Mersmann HJ, Smith EO, Britain KG. Stearoyl-coenzyme A desaturase gene expression during growth in adipose tissue from obese and crossbred pigs. J Anim Sci. 1999;77: 1710–1716.

23. Zhou Y, Xu Z, Wang L, Ling D, Nong Q, Xie J, et al. Cold exposure induces depot-specific alterations in fatty acid composition and transcriptional profile in adipose tissues of pigs. Front Endocrinol (Lausanne). 2022;13: 827523.

24. Renaville B, Bacciu N, Lanzoni M, Corazzin M, Piasentier E. Polymorphism of fat metabolism genes as candidate markers for meat quality and production traits in heavy pigs. Meat Sci. 2015;110: 220–223.

Supporting information

S1 Table. Primers for RT-PCR and cDNA sequence (A), genomic DNA sequence of exon 5 (B), c.267A>C genotyping (C), and qRT-PCR (D) in *SCD*.

Author Contributions

Conceptualization: Yuichi Koshiishi, Kenta Wada.

Data curation: Yuichi Koshiishi.

Investigation: Yuichi Koshiishi, Suzuka Moriya, Yuki Ishida, Hiromitsu Ochiai.

Formal analysis: Yuichi Koshiishi, Suzuka Moriya.

Supervision: Kenta Wada.

Writing – original draft: Yuichi Koshiishi, Kenta Wada.

Writing – review & editing: Yuichi Koshiishi, Kenta Wada, Suzuka Moriya, Yuki Ishida, and Hiromitsu Ochiai.

Ethics statement

All procedures involving animals met the guidelines described in "The Proper Conduct of Animal Experiments," proposed by the Science Council of Japan and were approved by the Ethical Care and Use of Animals Committee at the Tokyo University of Agriculture (approval number: 270049, 280002, 290096, 300126, and 2019109). The study was carried out in compliance with the ARRIVE guidelines.

Conflicts of interest

The authors declare no conflict of interest.

Data availability statement

All relevant data are within the manuscript and its Supporting Information files.

Click here to access/download;Figure;Fig1.tif

Koshiishi et al. Figure 1

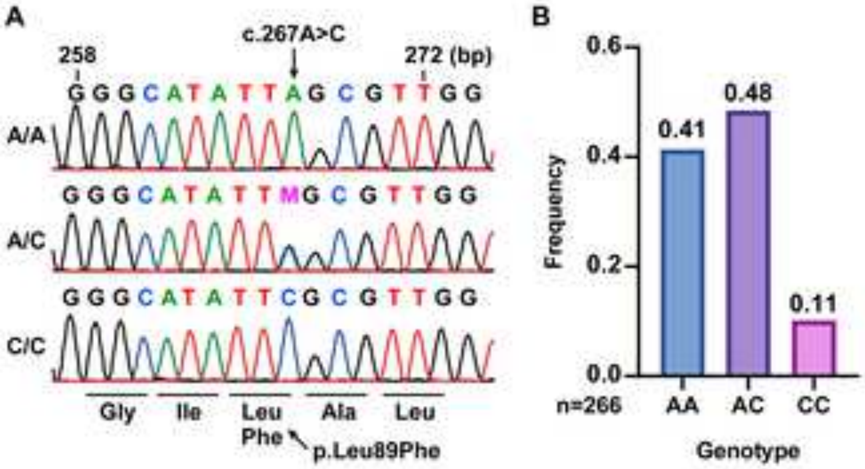

Koshiishi et al. Figure 2

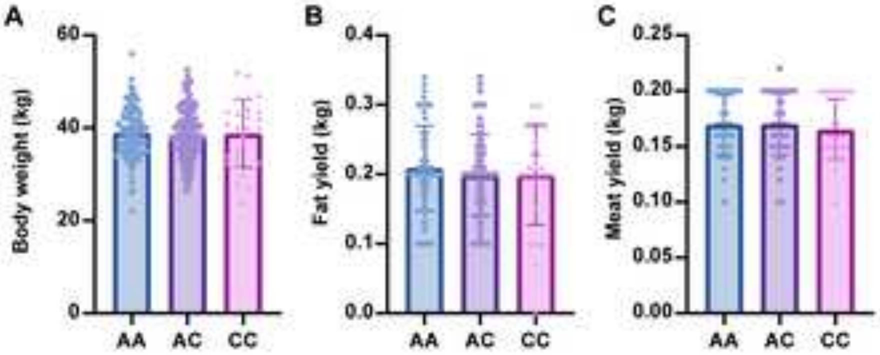

Click here to access/download;Figure;Fig3.tif

Koshiishi et al. Figure 3

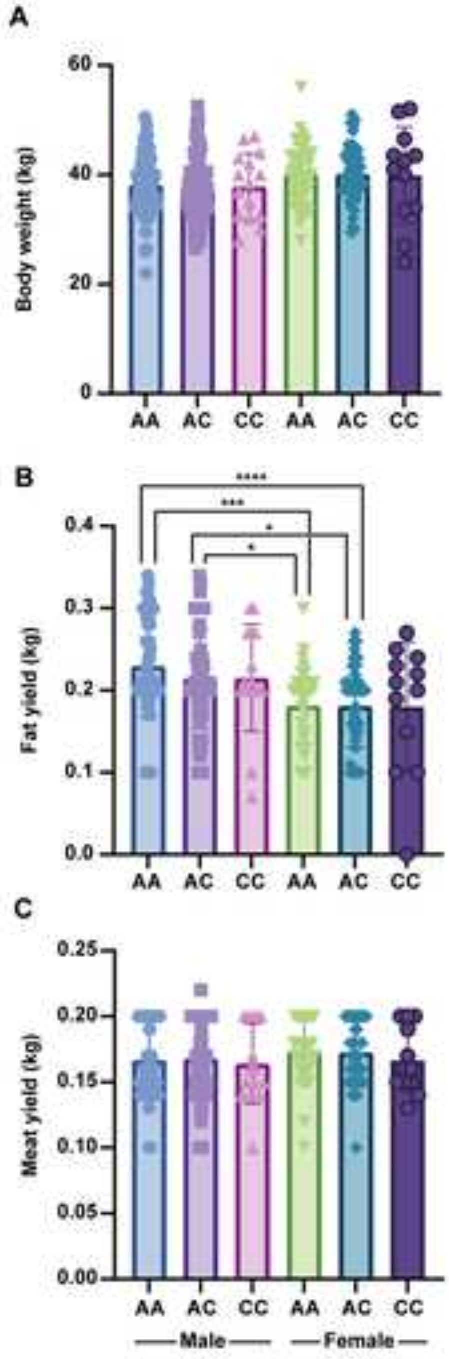

Click here to access/download;Figure;Fig4.tif

Koshiishi et al. Figure 4

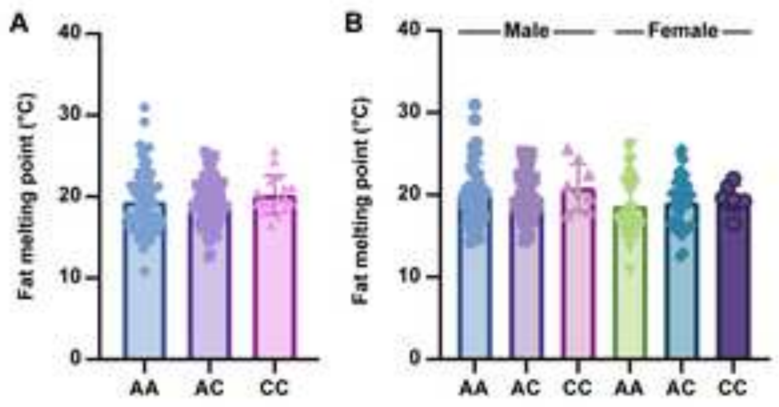

Click here to access/download;Figure;Fig5.tif

Koshiishi et al. Figure 5

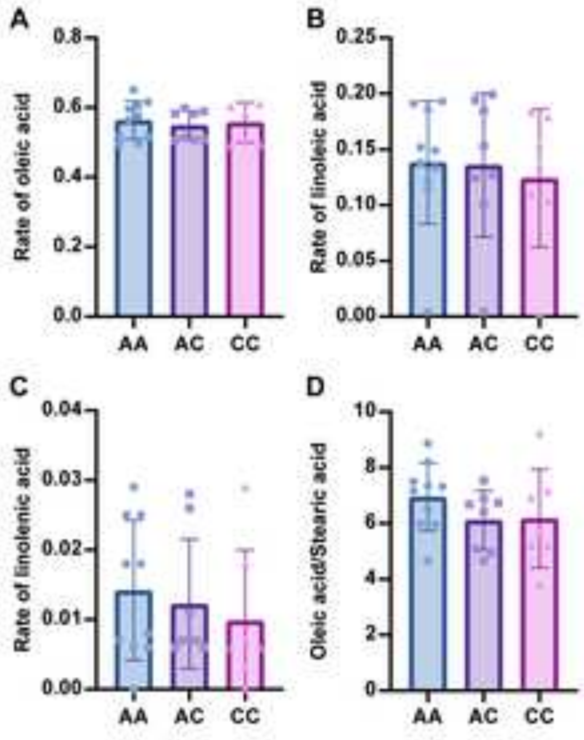

Koshiishi et al. Figure 6

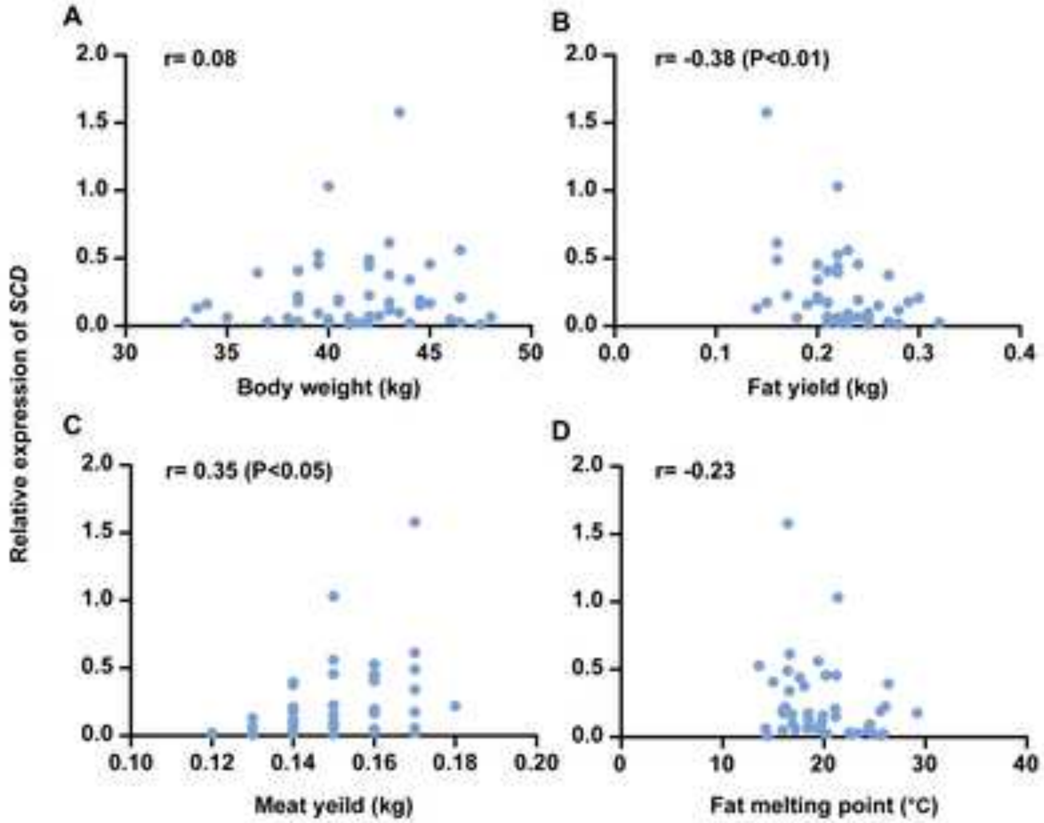

Click here to access/download
Supporting Information
S1_Table.xlsx
