# Supplementary material for: A polymorphism and expression levels of SCD and their relationships to production traits in a Japanese farmed emu population
Source: PLoS One. 2026 Jun 25;21(6):e0352695. doi: 10.1371/journal.pone.0352695 (PMC13298733; doi:10.1371/journal.pone.0352695)
Supplement: Attachment — Submitted filename: Reviewer comments.docx [file pone.0352695.s003.docx]

**Reviewer Comments**

**ABSTRACT:**

The abstract emphasizes the study's significance, objectives, findings, and conclusions. The sentence stating that "However, the expression levels of SCD transcripts were negatively correlated with fat content (P < 0.01) and positively correlated with meat yield (P < 0.05), suggesting that increased SCD transcript levels in subcutaneous fat are associated with reduced fat content and higher meat production in emus" should be removed, as the study's focus and results do not directly support this conclusion.

**Line 62-22**

It should be clarified that although progeny testing can be time-consuming and expensive, it is considered highly accurate because its results reflect the combined genetic influence on quantitative traits such as meat yield, fat content, and fatty acid profile. In contrast, molecular genetic improvement is reliable only when the markers used are universal and applicable across various environments and management systems.

**Line 74-79**

It should be clarified that when associating genotype with production traits, the environmental conditions of emu rearing used in this study must be explicitly stated. The impact of the c.267 A>C substitution mechanism on SCD may vary across environments and management systems. Therefore, to provide a more comprehensive understanding, the research design should include testing across multiple environments and management systems.

**Lines 83-91**

It should be clarified that:

1. The information on rearing management, diet, and the origin of the reared emus, based on Koshiishi et al. 2022, is inaccurate because the data for this study were collected between 2015 and 2017, whereas the referenced study is from 2022. Including a brief note on the environmental conditions during data collection would improve clarity.
2. The number of datasets for male and female emus in the 266 samples should be increased.
3. A short explanation of how the melting point and adipose tissue fat composition data were obtained is recommended.
4. An explanation is needed for why 168 datasets were used for melting point analysis, whereas only 25 datasets were used for fatty acid composition.

**Lines 140-143**

1. The data analysis method should be explained clearly. Are the data sets for males and females analyzed separately?
2. An explanation is needed for why ANOVA and T-tests were performed using different approaches.
3. Why was an equilibrium test not conducted for the observed genotypes? Performing this test is essential to determine whether the gene allele frequencies at the emu farm are in equilibrium. This information would help discuss the results.

**Line 167-175**

Further clarification is needed in the Discussion section:

1. When analyzing this association, were male and female data examined separately or pooled together?
2. It is important to consider whether the findings could be affected by various factors, such as (1) the relatively small dataset; (2) allele frequencies that may not be be in equilibrium due to factors like selection, mutation, migration, or genetic drift; and (3) the large standard deviations (SD) observed in mean body weight, fat yield, and meat yield, which could result from measurement errors or individual variability within the population. These points should be discussed further.

**Lines 182-183**

These outcomes vary by method. The method should specify the amount of data utilized.

**Lines 183-202**

It should be clarified whether the relatively small data set affected the results, which showed no significant differences between genotypes in both males and females.

**Lines 214-217 and 219-222**

It is better to omit the term "higher or lower tendency" and instead focus on whether the difference is statistically significant, basing the discussion solely on the statistical results.

**Lines 231-241**

The results of this study depend on the sample size used. Therefore, the discussion section should clarify whether the processed data included male and female samples or was analyzed separately. If combined, were the data sets comparable or significantly different? The varying data sizes for males and females could affect the results.

**Lines 269-274**

It is best to compare only poultry research results, as comparing poultry and mammalian studies can introduce bias due to their different metabolisms and growth patterns. In the discussion, the author mainly explains the SCD gene and the factors suspected to influence it. It would be helpful to add an explanation that quantitative traits such as body weight, fatness, and fatty acid composition are influenced by multiple genes working together to express a trait in specific environments. Therefore, it’s important to discuss the roles of other genes in the SCD gene network to determine whether selecting only the SCD gene as the factor influencing body weight, fatness, and fatty acid composition in emus is appropriate or whether including other genes would strengthen the results.

**Lines 320-321**

The sentence "However, the expression levels of SCD transcripts were negatively correlated with fat content (P < 0.01) and positively correlated with meat yield (P < 0.05), suggests that increased SCD transcript levels in subcutaneous fat are associated with reduced fat content and higher meat production in emus" should be omitted because the research methods and design do not support this claim.

**Lines 332-403**

The author should focus solely on research findings related to poultry, as poultry metabolism differs from that of non-avian animals, especially mammals, with variations in growth processes and stages.

**Figures**

It is recommended to improve the clarity and sharpness of the images, as much of the text within the figure is hard to read.

**Other remarks**

1. To the best of my knowledge, this publication has not previously used emus as research subjects or examined the SCD gene.

2. The research has been approved by the research ethics committee of the Science Council of Japan and the Ethical Care and Use of Animals Committee at Tokyo University of Agriculture (approval numbers: 270049, 280002, 290096, 300126, and 2019109).

3. To my knowledge, the research was conducted according to approved protocols and did not violate publication ethics.
