# Supplementary material for: A polymorphism and expression levels of SCD and their relationships to production traits in a Japanese farmed emu population
Source: PLoS One. 2026 Jun 25;21(6):e0352695. doi: 10.1371/journal.pone.0352695 (PMC13298733; doi:10.1371/journal.pone.0352695)
Supplement: Attachment — Submitted filename: Koshiishi_et_al_Response_Letter_v2.docx [file pone.0352695.s006.docx]

**Dear Editor,**

1. Please ensure that your manuscript meets PLOS ONE's style requirements, including those for file naming.

**Response:** Thank you for your comment. We revised the manuscript to meet the style requirements of *PLOS ONE*  (highlighted by green), including the file naming format.

2. Please provide the name of the slaughter house.

**Response:** Thank you for your comment. We added the name of the slaughterhouse to the manuscript (Lines 90–91, highlighted by green).

In addition, several minor errors in the manuscript were corrected and highlighted in green.

**Dear reviewer #1,**

Thank you for your positive and valuable comments on our manuscript. We have revised the manuscript according to your suggestions. Revised sentences are highlighted in light blue in the revised manuscript. We hope that the revised version is now suitable for publication.

1. **ABSTRACT:**

The abstract emphasizes the study's significance, objectives, findings, and conclusions. The sentence stating that "However, the expression levels of SCD transcripts were negatively correlated with fat content (P < 0.01) and positively correlated with meat yield (P < 0.05), suggesting that increased SCD transcript levels in subcutaneous fat are associated with reduced fat content and higher meat production in emus" should be removed, as the study's focus and results do not directly support this conclusion.

**Response:** Thank you for your comment. We agree this statement lacks support from our study design and results. We have removed it from the Abstract.

1. **Line 62-22**

It should be clarified that although progeny testing can be time-consuming and expensive, it is considered highly accurate because its results reflect the combined genetic influence on quantitative traits such as meat yield, fat content, and fatty acid profile. In contrast, molecular genetic improvement is reliable only when the markers used are universal and applicable across various environments and management systems.

**Response:** Thank you for your important comments. We added sentences explaining the accuracy of the progeny test to estimate quantitative traits in animals and noted that progeny testing is difficult in emu due to their breeding system. In addition, we added a statement on molecular genetic approach limitations, noting that marker-based selection is reliable only across consistent environments and management systems (Lines 62-68).

1. **Line 74-79**

It should be clarified that when associating genotype with production traits, the environmental conditions of emu rearing used in this study must be explicitly stated. The impact of the c.267 A>C substitution mechanism on SCD may vary across environments and management systems. Therefore, to provide a more comprehensive understanding, the research design should include testing across multiple environments and management systems.

**Response:** Thank you for your valuable comment. We agree environmental conditions are crucial when evaluating genotype-production trait associations. We added the description of rearing conditions in the Materials and Methods section to clarify the environmental background. We also noted in the Discussion that c.267A>C substitution effects may vary with environmental and management conditions and acknowledged that further studies across multiple environments and populations are needed to validate findings. We added sentences explaining the rearing conditions in the Materials and Methods section (Lines 88-91) and the importance of environmental effects in the Discussion section (Line 307-312 and 323-326).

1. **Lines 83-91**

It should be clarified that:

1. The information on rearing management, diet, and the origin of the reared emus, based on Koshiishi et al. 2022, is inaccurate because the data for this study were collected between 2015 and 2017, whereas the referenced study is from 2022. Including a brief note on the environmental conditions during data collection would improve clarity.

**Response:** Thank you for your comment. We apologize for the lack of clarity. Our samples are from a population described in Koshiishi et al. (2022), which included emus slaughtered between 2014 and 2018. We obtained genomic DNA from individuals slaughtered between 2015 and 2017, clarified in the revised manuscript (Lines 88-91). We also added a description of rearing conditions (diet, management) during data collection in the Materials and Methods section (Lines 105-110).

2. The number of datasets for male and female emus in the 266 samples should be increased.

**Response:** Thank you for your comment. We acknowledge that male and female datasets are limited and unbalanced. Sample availability prevented dataset expansion. We clarified individual counts by sex in the revised manuscript (Lines 91-92) and noted in the Discussion noting that larger, more balanced datasets are necessary to validate findings (Lines 311-312 and 325-326).

3. A short explanation of how the melting point and adipose tissue fat composition data were obtained is recommended.

**Response:** Thank you for your suggestion. We have added descriptions of fat melting point and fatty acid composition measurement methods in the Materials and Methods section (Lines 94-110).

4. An explanation is needed for why 168 datasets were used for melting point analysis, whereas only 25 datasets were used for fatty acid composition.

**Response:** Thank you for your comment. The dataset size difference (fat melting point n = 168 vs. fatty acid composition n = 25) reflects technical and resource constraints. Fatty acid analysis requires several grams of adipose tissue per individual, unavailable for all samples. Additionally, high cost and labor-intensity restricted analysis to a subset. Individuals were selected to represent fat melting point ranges (low, intermediate, and high) to minimize bias. We clarified this in the Materials and Methods section (Lines 99-105) and noted in the Discussion that the smaller sample size may limit the statistical power; larger datasets needed for validation (Lines 311).

1. **Lines 140-143**
1. The data analysis method should be explained clearly. Are the data sets for males and females analyzed separately?

**Response:** Thank you for your comment. The data for fatty acid composition were analyzed using combined datasets for males and females due to the limited sample size. We have clarified this point in the Materials and Methods section (Lines 160-162).

2. An explanation is needed for why ANOVA and T-tests were performed using different approaches.

**Response:** Thank you for your comment. We used ANOVA to compare production traits among three *SCD* genotypes (AA, AC, and CC) and t-tests for two group comparisons, such as sex differences. Different statistical approaches matched the number of groups compared. We clarified this in the Materials and Methods section (Lines 162-165).

3. **Why was an equilibrium test not conducted for the observed genotypes? Performing this test is essential to determine whether the gene allele frequencies at the emu farm are in equilibrium. This information would help discuss the results.**

**Response:** Thank you for your valuable suggestion. We performed a Hardy–Weinberg equilibrium (HWE) test and confirmed that genotype frequencies did not significantly deviate from equilibrium. We added the HWE test results in the Results section (Lines 182-184).

1. **Line 167-175**

Further clarification is needed in the Discussion section:

1. When analyzing this association, were male and female data examined separately or pooled together?

**Response:** Thank you for your comment. The association between genotypes and production traits was analyzed both separately for males and females and using a combined dataset. Sex-specific analyses were performed to account for potential sex-related differences, while combined analyses were conducted to evaluate overall genotype effects. In contrast, fatty acid composition was analyzed using combined datasets due to the limited sample size and because no significant sex differences were observed in these traits in our previous study. We have clarified this point in the Materials and Methods section (Lines 99-105).

2. **It is important to consider whether the findings could be affected by various factors, such as (1) the relatively small dataset; (2) allele frequencies that may not be be in equilibrium due to factors like selection, mutation, migration, or genetic drift; and (3) the large standard deviations (SD) observed in mean body weight, fat yield, and meat yield, which could result from measurement errors or individual variability within the population. These points should be discussed further.**

**Response:** Thank you for your valuable comment. We agree that several factors may have influenced the study results. We expanded the Discussion section to address these points. First, we acknowledge that the relatively small sample size, particularly in fatty acid composition analysis, may have limited the statistical power to detect genotype differences. Second, we performed a Hardy-Weinberg equilibrium (HWE) test and confirmed that genotype frequencies did not significantly deviate from equilibrium, indicating no substantial distortion of allele frequencies in the studied population. Third, the large standard deviations observed in some production traits may reflect population variability and environmental influences. We have added these considerations to the revised manuscript (Lines 301-307).

1. **Lines 182-183**

These outcomes vary by method. The method should specify the amount of data utilized.

**Response:** Thank you for your comment. We have clarified sample sizes for each analysis in the Materials and Methods section.

1. **Lines 183-202**

It should be clarified whether the relatively small data set affected the results, which showed no significant differences between genotypes in both males and females.

**Response:** Thank you for your comment. We acknowledge that the relatively small sample size may have affected the statistical power of the analyses, potentially limiting the detection of significant differences between genotypes in both males and females. We have added this point to the Discussion section and noted that further studies with larger sample sizes are required to validate these findings (Lines 301-312).

1. **Lines 214-217 and 219-222**

It is better to omit the term "higher or lower tendency" and instead focus on whether the difference is statistically significant, basing the discussion solely on the statistical results.

**Response:** Thank you for your comment. We agree that terms such as 'higher' or 'lower tendency' may be misleading. We have revised the manuscript to remove such expressions and base the discussion solely on statistically significant results.

1. **Lines 231-241**

The results of this study depend on the sample size used. Therefore, the discussion section should clarify whether the processed data included male and female samples or was analyzed separately. If combined, were the data sets comparable or significantly different? The varying data sizes for males and females could affect the results.

**Response:** Thank you for your valuable comment. Production traits were analyzed separately for males and females and as a combined dataset. In contrast, fatty acid composition and gene expression analyses used pooled male and female samples due to limited sample size. Our previous study showed no significant sex differences in fatty acid composition except linolenic acid content, which was significantly higher in males than females (P < 0.01). However, no significant association was observed between *SCD* genotype and linolenic acid content in males. We have added these explanations to the Discussion section and clarified the potential influence of sample size differences between sexes (Lines 302-305).

1. **Lines 269-274**

It is best to compare only poultry research results, as comparing poultry and mammalian studies can introduce bias due to their different metabolisms and growth patterns. In the discussion, the author mainly explains the SCD gene and the factors suspected to influence it. It would be helpful to add an explanation that quantitative traits such as body weight, fatness, and fatty acid composition are influenced by multiple genes working together to express a trait in specific environments. Therefore, it’s important to discuss the roles of other genes in the SCD gene network to determine whether selecting only the SCD gene as the factor influencing body weight, fatness, and fatty acid composition in emus is appropriate or whether including other genes would strengthen the results.

**Response:** Thank you for your valuable comment. We agree that production traits such as body weight and fatty acid composition are complex quantitative traits influenced by multiple genes and environmental factors. Accordingly, we have revised the Discussion to include this perspective and to clarify that *SCD* represents only one of the potential genetic factors involved. We have also reduced overemphasis on *SCD* and added a brief discussion on the potential involvement of other genes in the regulation of these traits (Line 307-312).

1. **Lines 320-321**

The sentence "However, the expression levels of SCD transcripts were negatively correlated with fat content (P < 0.01) and positively correlated with meat yield (P < 0.05), suggests that increased SCD transcript levels in subcutaneous fat are associated with reduced fat content and higher meat production in emus" should be omitted because the research methods and design do not support this claim.

**Response:** Thank you for your valuable comment. We agree this statement could be interpreted as implying a functional relationship not fully supported by our study design and results. Therefore, we have revised the Conclusion to remove this implication, eliminated expressions such as 'tended to be associated,' and present results strictly based on statistically supported findings (Lines 330-333).

1. **Lines 332-403**

The author should focus solely on research findings related to poultry, as poultry metabolism differs from that of non-avian animals, especially mammals, with variations in growth processes and stages.

**Response:** Thank you for your valuable comment. We agree that metabolic differences between avian and non-avian species, particularly mammals, should be carefully considered. Accordingly, we have revised the Discussion to emphasize findings from poultry studies (Lines 293-301).

1. **Figures**

It is recommended to improve the clarity and sharpness of the images, as much of the text within the figure is hard to read.

**Response:** Thank you for your comment. We have replaced the figures with higher-resolution images and improved the readability of the text within the figures.

**Dear reviewer #2,**

Thank you for your valuable comments on our manuscript. We have revised the manuscript according to your suggestions. Revised sentences are highlighted in yellow in the revised manuscript. We hope that the revised version is now suitable for publication.

・Overall:

Thank you for your careful review and helpful suggestions regarding the manuscript. We have revised it accordingly.

・L23: Is predicted to become or is?

**Response:** Thank you for your suggestion. We revised the sentence as follows: The emu is a novel poultry species producing meat, eggs, and fat (Line 23).

・L55-56: What is the meaning of this sentence? Why?

**Response:** Thank you for your comment. We revised this sentence to clarify that the reproductive characteristics of emus, including polyandry and male egg incubation, make accurate pedigree recording difficult for selective breeding (Line 54).

・L142: What about correlations?

**Response:** Thank you for your comment. We added a description of the correlation analysis method in the Materials and Methods section. Correlations between production traits and *SCD* expression levels were evaluated using Pearson’s correlation coefficient (Lines 165-166).

・L288: Incomplete sentence

**Response:** Thank you for your comment. We revised the Discussion section extensively according to the reviewers’ comments, and the incomplete sentence was removed in the revised manuscript.
