# Supplementary material for: A polymorphism and expression levels of SCD and their relationships to production traits in a Japanese farmed emu population
Source: PLoS One. 2026 Jun 25;21(6):e0352695. doi: 10.1371/journal.pone.0352695 (PMC13298733; doi:10.1371/journal.pone.0352695)
Supplement: Peer review history

# Peer review: Decision Letter 0

**DOI:** 10.1371/journal.pone.0352695.r001

**Authors:** Abbas Farmany

15 Apr 2026

PONE-D-26-06317

A polymorphism and expression levels of SCD and their relationships to production traits in a Japanese farmed emu population

PLOS One

Dear Dr. Wada,

Thank you for submitting your manuscript to PLOS ONE. After careful consideration, we feel that it has merit but does not fully meet PLOS ONE’s publication criteria as it currently stands. Therefore, we invite you to submit a revised version of the manuscript that addresses the points raised during the review process.

Please submit your revised manuscript by May 30 2026 11:59PM. If you will need more time than this to complete your revisions, please reply to this message or contact the journal office at plosone@plos.org. When you're ready to submit your revision, log on to https://www.editorialmanager.com/pone/ and select the 'Submissions Needing Revision' folder to locate your manuscript file.

Please include the following items when submitting your revised manuscript:

- A letter that responds to each point raised by the academic editor and reviewer(s). You should upload this letter as a separate file labeled 'Response to Reviewers'.
- A marked-up copy of your manuscript that highlights changes made to the original version. You should upload this as a separate file labeled 'Revised Manuscript with Track Changes'.
- An unmarked version of your revised paper without tracked changes. You should upload this as a separate file labeled 'Manuscript'.

If you would like to make changes to your financial disclosure, please include your updated statement in your cover letter. Guidelines for resubmitting your figure files are available below the reviewer comments at the end of this letter.

If applicable, we recommend that you deposit your laboratory protocols in protocols.io to enhance the reproducibility of your results. Protocols.io assigns your protocol its own identifier (DOI) so that it can be cited independently in the future. For instructions see: https://journals.plos.org/plosone/s/submission-guidelines#loc-laboratory-protocols. Additionally, PLOS ONE offers an option for publishing peer-reviewed Lab Protocol articles, which describe protocols hosted on protocols.io. Read more information on sharing protocols at https://plos.org/protocols?utm_medium=editorial-email&utm_source=authorletters&utm_campaign=protocols.

As the corresponding author, your ORCID iD is verified in the submission system and will appear in the published article. PLOS supports the use of ORCID, and we encourage all coauthors to register for an ORCID iD and use it as well. Please encourage your coauthors to verify their ORCID iD within the submission system before final acceptance, as unverified ORCID iDs will not appear in the published article. *Only*  the individual author can complete the verification step; PLOS staff *cannot*  verify ORCID iDs on behalf of authors.

We look forward to receiving your revised manuscript.

Kind regards,

Abbas Farmany

Academic Editor

PLOS One

Journal requirements:

When submitting your revision, we need you to address these additional requirements.

1. Please ensure that your manuscript meets PLOS ONE's style requirements, including those for file naming. The PLOS ONE style templates can be found at

https://journals.plos.org/plosone/s/file?id=wjVg/PLOSOne_formatting_sample_main_body.pdf and

https://journals.plos.org/plosone/s/file?id=ba62/PLOSOne_formatting_sample_title_authors_affiliations.pdf

2. Please provide the name of the slaughter house.

If the reviewer comments include a recommendation to cite specific previously published works, please review and evaluate these publications to determine whether they are relevant and should be cited. There is no requirement to cite these works unless the editor has indicated otherwise.

[Note: HTML markup is below. Please do not edit.]

Reviewers' comments:

Reviewer's Responses to Questions

**Comments to the Author**

1. Is the manuscript technically sound, and do the data support the conclusions?

The manuscript must describe a technically sound piece of scientific research with data that supports the conclusions. Experiments must have been conducted rigorously, with appropriate controls, replication, and sample sizes. The conclusions must be drawn appropriately based on the data presented.

Reviewer #1: Partly

Reviewer #2: Yes

**********

2. Has the statistical analysis been performed appropriately and rigorously?

Reviewer #1: No

Reviewer #2: Yes

**********

3. Have the authors made all data underlying the findings in their manuscript fully available?

The PLOS Data policy requires authors to make all data underlying the findings described in their manuscript fully available without restriction, with rare exception (please refer to the Data Availability Statement in the manuscript PDF file). The data should be provided as part of the manuscript or its supporting information, or deposited to a public repository. For example, in addition to summary statistics, the data points behind means, medians and variance measures should be available. If there are restrictions on publicly sharing data—e.g. participant privacy or use of data from a third party—those must be specified.

Reviewer #1: No

Reviewer #2: No

**********

4. Is the manuscript presented in an intelligible fashion and written in standard English?

PLOS ONE does not copyedit accepted manuscripts, so the language in submitted articles must be clear, correct, and unambiguous. Any typographical or grammatical errors should be corrected at revision, so please note any specific errors here.

Reviewer #1: Yes

Reviewer #2: Yes

**********

5. Review Comments to the Author

Please use the space provided to explain your answers to the questions above. You may also include additional comments for the author, including concerns about dual publication, research ethics, or publication ethics. (Please upload your review as an attachment if it exceeds 20,000 characters)

Reviewer #1: Reviewer Comments

ABSTRACT:

The abstract emphasizes the study's significance, objectives, findings, and conclusions. The sentence stating that "However, the expression levels of SCD transcripts were negatively correlated with fat content (P < 0.01) and positively correlated with meat yield (P < 0.05), suggesting that increased SCD transcript levels in subcutaneous fat are associated with reduced fat content and higher meat production in emus" should be removed, as the study's focus and results do not directly support this conclusion.

Line 62-22

It should be clarified that although progeny testing can be time-consuming and expensive, it is considered highly accurate because its results reflect the combined genetic influence on quantitative traits such as meat yield, fat content, and fatty acid profile. In contrast, molecular genetic improvement is reliable only when the markers used are universal and applicable across various environments and management systems.

Line 74-79

It should be clarified that when associating genotype with production traits, the environmental conditions of emu rearing used in this study must be explicitly stated. The impact of the c.267 A>C substitution mechanism on SCD may vary across environments and management systems. Therefore, to provide a more comprehensive understanding, the research design should include testing across multiple environments and management systems.

Lines 83-91

It should be clarified that:

1.The information on rearing management, diet, and the origin of the reared emus, based on Koshiishi et al. 2022, is inaccurate because the data for this study were collected between 2015 and 2017, whereas the referenced study is from 2022. Including a brief note on the environmental conditions during data collection would improve clarity.

2.The number of datasets for male and female emus in the 266 samples should be increased.

3.A short explanation of how the melting point and adipose tissue fat composition data were obtained is recommended.

4.An explanation is needed for why 168 datasets were used for melting point analysis, whereas only 25 datasets were used for fatty acid composition.

Lines 140-143

1.The data analysis method should be explained clearly. Are the data sets for males and females analyzed separately?

2.An explanation is needed for why ANOVA and T-tests were performed using different approaches.

3.Why was an equilibrium test not conducted for the observed genotypes? Performing this test is essential to determine whether the gene allele frequencies at the emu farm are in equilibrium. This information would help discuss the results.

Line 167-175

Further clarification is needed in the Discussion section:

1.When analyzing this association, were male and female data examined separately or pooled together?

2.It is important to consider whether the findings could be affected by various factors, such as (1) the relatively small dataset; (2) allele frequencies that may not be be in equilibrium due to factors like selection, mutation, migration, or genetic drift; and (3) the large standard deviations (SD) observed in mean body weight, fat yield, and meat yield, which could result from measurement errors or individual variability within the population. These points should be discussed further.

Lines 182-183

These outcomes vary by method. The method should specify the amount of data utilized.

Lines 183-202

It should be clarified whether the relatively small data set affected the results, which showed no significant differences between genotypes in both males and females.

Lines 214-217 and 219-222

It is better to omit the term "higher or lower tendency" and instead focus on whether the difference is statistically significant, basing the discussion solely on the statistical results.

Lines 231-241

The results of this study depend on the sample size used. Therefore, the discussion section should clarify whether the processed data included male and female samples or was analyzed separately. If combined, were the data sets comparable or significantly different? The varying data sizes for males and females could affect the results.

Lines 269-274

It is best to compare only poultry research results, as comparing poultry and mammalian studies can introduce bias due to their different metabolisms and growth patterns. In the discussion, the author mainly explains the SCD gene and the factors suspected to influence it. It would be helpful to add an explanation that quantitative traits such as body weight, fatness, and fatty acid composition are influenced by multiple genes working together to express a trait in specific environments. Therefore, it’s important to discuss the roles of other genes in the SCD gene network to determine whether selecting only the SCD gene as the factor influencing body weight, fatness, and fatty acid composition in emus is appropriate or whether including other genes would strengthen the results.

Lines 320-321

The sentence "However, the expression levels of SCD transcripts were negatively correlated with fat content (P < 0.01) and positively correlated with meat yield (P < 0.05), suggests that increased SCD transcript levels in subcutaneous fat are associated with reduced fat content and higher meat production in emus" should be omitted because the research methods and design do not support this claim.

Lines 332-403

The author should focus solely on research findings related to poultry, as poultry metabolism differs from that of non-avian animals, especially mammals, with variations in growth processes and stages.

Figures

It is recommended to improve the clarity and sharpness of the images, as much of the text within the figure is hard to read.

Other remarks

1. To the best of my knowledge, this publication has not previously used emus as research subjects or examined the SCD gene.

2. The research has been approved by the research ethics committee of the Science Council of Japan and the Ethical Care and Use of Animals Committee at Tokyo University of Agriculture (approval numbers: 270049, 280002, 290096, 300126, and 2019109).

3. To my knowledge, the research was conducted according to approved protocols and did not violate publication ethics.

Reviewer #2: This article provides information on the effects of SCD polymorphism and expression levels on production traits in a Japanese farmed emu population. It is in general appropriately organized, carried out and written, however there are some points that should be corrected or clarified. Please check comments and corrections in the attached file.

**********

6. PLOS authors have the option to publish the peer review history of their article (what does this mean?). If published, this will include your full peer review and any attached files.

If you choose “no”, your identity will remain anonymous but your review may still be made public.

**Do you want your identity to be public for this peer review?**  For information about this choice, including consent withdrawal, please see our Privacy Policy.

Reviewer #1: No

Reviewer #2: No

**********

[NOTE: If reviewer comments were submitted as an attachment file, they will be attached to this email and accessible via the submission site. Please log into your account, locate the manuscript record, and check for the action link "View Attachments". If this link does not appear, there are no attachment files.]

To ensure your figures meet our technical requirements, please review our figure guidelines: https://journals.plos.org/plosone/s/figures

You may also use PLOS’s free figure tool, NAAS, to help you prepare publication quality figures: https://journals.plos.org/plosone/s/figures#loc-tools-for-figure-preparation.

NAAS will assess whether your figures meet our technical requirements by comparing each figure against our figure specifications.

Attachment Submitted filename: Reviewer comments.docx

Attachment Submitted filename: PONE-D-26-06317_reviewer.pdf

# Author comment: Author response to Decision Letter 1

**DOI:** 10.1371/journal.pone.0352695.r002

6 Jun 2026

Dear Editor,

1. Please ensure that your manuscript meets PLOS ONE's style requirements, including those for file naming.

Response: Thank you for your comment. We revised the manuscript to meet the style requirements of PLOS ONE, including the file naming format.

2. Please provide the name of the slaughter house.

Response: Thank you for your comment. We added the name of the slaughterhouse to the manuscript (Lines 90–91, highlighted by green).

Dear reviewer #1,

Thank you for your positive and valuable comments on our manuscript. We have revised the manuscript according to your suggestions. Revised sentences are highlighted in light blue in the revised manuscript. We hope that the revised version is now suitable for publication.

① ABSTRACT:

The abstract emphasizes the study's significance, objectives, findings, and conclusions. The sentence stating that "However, the expression levels of SCD transcripts were negatively correlated with fat content (P < 0.01) and positively correlated with meat yield (P < 0.05), suggesting that increased SCD transcript levels in subcutaneous fat are associated with reduced fat content and higher meat production in emus" should be removed, as the study's focus and results do not directly support this conclusion.

Response: Thank you for your comment. We agree this statement lacks support from our study design and results. We have removed it from the Abstract.

② Line 62-22

It should be clarified that although progeny testing can be time-consuming and expensive, it is considered highly accurate because its results reflect the combined genetic influence on quantitative traits such as meat yield, fat content, and fatty acid profile. In contrast, molecular genetic improvement is reliable only when the markers used are universal and applicable across various environments and management systems.

Response: Thank you for your important comments. We added sentences explaining the accuracy of the progeny test to estimate quantitative traits in animals and noted that progeny testing is difficult in emu due to their breeding system. In addition, we added a statement on molecular genetic approach limitations, noting that marker-based selection is reliable only across consistent environments and management systems (Lines 62-68).

③ Line 74-79

It should be clarified that when associating genotype with production traits, the environmental conditions of emu rearing used in this study must be explicitly stated. The impact of the c.267 A>C substitution mechanism on SCD may vary across environments and management systems. Therefore, to provide a more comprehensive understanding, the research design should include testing across multiple environments and management systems.

Response: Thank you for your valuable comment. We agree environmental conditions are crucial when evaluating genotype-production trait associations. We added the description of rearing conditions in the Materials and Methods section to clarify the environmental background. We also noted in the Discussion that c.267A>C substitution effects may vary with environmental and management conditions and acknowledged that further studies across multiple environments and populations are needed to validate findings. We added sentences explaining the rearing conditions in the Materials and Methods section (Lines 88-91) and the importance of environmental effects in the Discussion section (Line 307-312 and 323-326).

④ Lines 83-91

It should be clarified that:

1. The information on rearing management, diet, and the origin of the reared emus, based on Koshiishi et al. 2022, is inaccurate because the data for this study were collected between 2015 and 2017, whereas the referenced study is from 2022. Including a brief note on the environmental conditions during data collection would improve clarity.

Response: Thank you for your comment. We apologize for the lack of clarity. Our samples are from a population described in Koshiishi et al. (2022), which included emus slaughtered between 2014 and 2018. We obtained genomic DNA from individuals slaughtered between 2015 and 2017, clarified in the revised manuscript (Lines 88-91). We also added a description of rearing conditions (diet, management) during data collection in the Materials and Methods section (Lines 105-110).

2. The number of datasets for male and female emus in the 266 samples should be increased.

Response: Thank you for your comment. We acknowledge that male and female datasets are limited and unbalanced. Sample availability prevented dataset expansion. We clarified individual counts by sex in the revised manuscript (Lines 91-92) and noted in the Discussion noting that larger, more balanced datasets are necessary to validate findings (Lines 311-312 and 325-326).

3. A short explanation of how the melting point and adipose tissue fat composition data were obtained is recommended.

Response: Thank you for your suggestion. We have added descriptions of fat melting point and fatty acid composition measurement methods in the Materials and Methods section (Lines 94-110).

4. An explanation is needed for why 168 datasets were used for melting point analysis, whereas only 25 datasets were used for fatty acid composition.

Response: Thank you for your comment. The dataset size difference (fat melting point n = 168 vs. fatty acid composition n = 25) reflects technical and resource constraints. Fatty acid analysis requires several grams of adipose tissue per individual, unavailable for all samples. Additionally, high cost and labor-intensity restricted analysis to a subset. Individuals were selected to represent fat melting point ranges (low, intermediate, and high) to minimize bias. We clarified this in the Materials and Methods section (Lines 99-105) and noted in the Discussion that the smaller sample size may limit the statistical power; larger datasets needed for validation (Lines 311).

⑤ Lines 140-143

1. The data analysis method should be explained clearly. Are the data sets for males and females analyzed separately?

Response: Thank you for your comment. The data for fatty acid composition were analyzed using combined datasets for males and females due to the limited sample size. We have clarified this point in the Materials and Methods section (Lines 160-162).

2. An explanation is needed for why ANOVA and T-tests were performed using different approaches.

Response: Thank you for your comment. We used ANOVA to compare production traits among three SCD genotypes (AA, AC, and CC) and t-tests for two group comparisons, such as sex differences. Different statistical approaches matched the number of groups compared. We clarified this in the Materials and Methods section (Lines 162-165).

3. Why was an equilibrium test not conducted for the observed genotypes? Performing this test is essential to determine whether the gene allele frequencies at the emu farm are in equilibrium. This information would help discuss the results.

Response: Thank you for your valuable suggestion. We performed a Hardy–Weinberg equilibrium (HWE) test and confirmed that genotype frequencies did not significantly deviate from equilibrium. We added the HWE test results in the Results section (Lines 182-184).

⑥ Line 167-175

Further clarification is needed in the Discussion section:

1. When analyzing this association, were male and female data examined separately or pooled together?

Response: Thank you for your comment. The association between genotypes and production traits was analyzed both separately for males and females and using a combined dataset. Sex-specific analyses were performed to account for potential sex-related differences, while combined analyses were conducted to evaluate overall genotype effects. In contrast, fatty acid composition was analyzed using combined datasets due to the limited sample size and because no significant sex differences were observed in these traits in our previous study. We have clarified this point in the Materials and Methods section (Lines 99-105).

2. It is important to consider whether the findings could be affected by various factors, such as (1) the relatively small dataset; (2) allele frequencies that may not be be in equilibrium due to factors like selection, mutation, migration, or genetic drift; and (3) the large standard deviations (SD) observed in mean body weight, fat yield, and meat yield, which could result from measurement errors or individual variability within the population. These points should be discussed further.

Response: Thank you for your valuable comment. We agree that several factors may have influenced the study results. We expanded the Discussion section to address these points. First, we acknowledge that the relatively small sample size, particularly in fatty acid composition analysis, may have limited the statistical power to detect genotype differences. Second, we performed a Hardy-Weinberg equilibrium (HWE) test and confirmed that genotype frequencies did not significantly deviate from equilibrium, indicating no substantial distortion of allele frequencies in the studied population. Third, the large standard deviations observed in some production traits may reflect population variability and environmental influences. We have added these considerations to the revised manuscript (Lines 301-307).

⑦ Lines 182-183

These outcomes vary by method. The method should specify the amount of data utilized.

Response: Thank you for your comment. We have clarified sample sizes for each analysis in the Materials and Methods section.

⑧ Lines 183-202

It should be clarified whether the relatively small data set affected the results, which showed no significant differences between genotypes in both males and females.

Response: Thank you for your comment. We acknowledge that the relatively small sample size may have affected the statistical power of the analyses, potentially limiting the detection of significant differences between genotypes in both males and females. We have added this point to the Discussion section and noted that further studies with larger sample sizes are required to validate these findings (Lines 301-312).

⑨ Lines 214-217 and 219-222

It is better to omit the term "higher or lower tendency" and instead focus on whether the difference is statistically significant, basing the discussion solely on the statistical results.

Response: Thank you for your comment. We agree that terms such as 'higher' or 'lower tendency' may be misleading. We have revised the manuscript to remove such expressions and base the discussion solely on statistically significant results.

⑩ Lines 231-241

The results of this study depend on the sample size used. Therefore, the discussion section should clarify whether the processed data included male and female samples or was analyzed separately. If combined, were the data sets comparable or significantly different? The varying data sizes for males and females could affect the results.

Response: Thank you for your valuable comment. Production traits were analyzed separately for males and females and as a combined dataset. In contrast, fatty acid composition and gene expression analyses used pooled male and female samples due to limited sample size. Our previous study showed no significant sex differences in fatty acid composition except linolenic acid content, which was significantly higher in males than females (P < 0.01). However, no significant association was observed between SCD genotype and linolenic acid content in males. We have added these explanations to the Discussion section and clarified the potential influence of sample size differences between sexes (Lines 302-305).

⑪ Lines 269-274

It is best to compare only poultry research results, as comparing poultry and mammalian studies can introduce bias due to their different metabolisms and growth patterns. In the discussion, the author mainly explains the SCD gene and the factors suspected to influence it. It would be helpful to add an explanation that quantitative traits such as body weight, fatness, and fatty acid composition are influenced by multiple genes working together to express a trait in specific environments. Therefore, it’s important to discuss the roles of other genes in the SCD gene network to determine whether selecting only the SCD gene as the factor influencing body weight, fatness, and fatty acid composition in emus is appropriate or whether including other genes would strengthen the results.

Response: Thank you for your valuable comment. We agree that production traits such as body weight and fatty acid composition are complex quantitative traits influenced by multiple genes and environmental factors. Accordingly, we have revised the Discussion to include this perspective and to clarify that SCD represents only one of the potential genetic factors involved. We have also reduced overemphasis on SCD and added a brief discussion on the potential involvement of other genes in the regulation of these traits (Line 307-312).

⑫ Lines 320-321

The sentence "However, the expression levels of SCD transcripts were negatively correlated with fat content (P < 0.01) and positively correlated with meat yield (P < 0.05), suggests that increased SCD transcript levels in subcutaneous fat are associated with reduced fat content and higher meat production in emus" should be omitted because the research methods and design do not support this claim.

Response: Thank you for your valuable comment. We agree this statement could be interpreted as implying a functional relationship not fully supported by our study design and results. Therefore, we have revised the Conclusion to remove this implication, eliminated expressions such as 'tended to be associated,' and present results strictly based on statistically supported findings (Lines 330-333).

⑬ Lines 332-403

The author should focus solely on research findings related to poultry, as poultry metabolism differs from that of non-avian animals, especially mammals, with variations in growth processes and stages.

Response: Thank you for your valuable comment. We agree that metabolic differences between avian and non-avian species, particularly mammals, should be carefully considered. Accordingly, we have revised the Discussion to emphasize findings from poultry studies (Lines 293-301).

⑭ Figures

It is recommended to improve the clarity and sharpness of the images, as much of the text within the figure is hard to read.

Response: Thank you for your comment. We have replaced the figures with higher-resolution images and improved the readability of the text within the figures.

Dear reviewer #2,

Thank you for your valuable comments on our manuscript. We have revised the manuscript according to your suggestions. Revised sentences are highlighted in yellow in the revised manuscript. We hope that the revised version is now suitable for publication.

・Overall:

Thank you for your careful review and helpful suggestions regarding the manuscript. We have revised it accordingly.

・L23: Is predicted to become or is?

Response: Thank you for your suggestion. We revised the sentence as follows: The emu is a novel poultry species producing meat, eggs, and fat (Line 23).

・L55-56: What is the meaning of this sentence? Why?

Response: Thank you for your comment. We revised this sentence to clarify that the reproductive characteristics of emus, including polyandry and male egg incubation, make accurate pedigree recording difficult for selective breeding (Line 54).

・L142: What about correlations?

Response: Thank you for your comment. We added a description of the correlation analysis method in the Materials and Methods section. Correlations between production traits and SCD expression levels were evaluated using Pearson’s correlation coefficient (Lines 165-166).

・L288: Incomplete sentence

Response: Thank you for your comment. We revised the Discussion section extensively according to the reviewers’ comments, and the incomplete sentence was removed in the revised manuscript.

Attachment Submitted filename: Koshiishi_et_al_Response_Letter_v2.docx

# Peer review: Decision Letter 1

**DOI:** 10.1371/journal.pone.0352695.r003

**Authors:** Abbas Farmany

14 Jun 2026

<p>A polymorphism and expression levels of SCD and their relationships to production traits in a Japanese farmed emu population

PONE-D-26-06317R1

Dear Dr. Wada,

We’re pleased to inform you that your manuscript has been judged scientifically suitable for publication and will be formally accepted for publication once it meets all outstanding technical requirements.

Within one week, you’ll receive an e-mail detailing the required amendments. When these have been addressed, you’ll receive a formal acceptance letter and your manuscript will be scheduled for publication.

An invoice will be generated when your article is formally accepted. Please note, if your institution has a publishing partnership with PLOS and your article meets the relevant criteria, all or part of your publication costs will be covered. Please make sure your user information is up-to-date by logging into Editorial Manager at Editorial Manager® and clicking the ‘Update My Information' link at the top of the page. For questions related to billing, please contact billing support.

If your institution or institutions have a press office, please notify them about your upcoming paper to help maximize its impact. If they’ll be preparing press materials, please inform our press team as soon as possible -- no later than 48 hours after receiving the formal acceptance. Your manuscript will remain under strict press embargo until 2 pm Eastern Time on the date of publication. For more information, please contact onepress@plos.org.

Kind regards,

Abbas Farmany

Academic Editor

PLOS One

Additional Editor Comments (optional):

Reviewers' comments:

Reviewer's Responses to Questions

**Comments to the Author**

1. If the authors have adequately addressed your comments raised in a previous round of review and you feel that this manuscript is now acceptable for publication, you may indicate that here to bypass the “Comments to the Author” section, enter your conflict of interest statement in the “Confidential to Editor” section, and submit your "Accept" recommendation.

Reviewer #1: All comments have been addressed

Reviewer #2: All comments have been addressed

**********

2. Is the manuscript technically sound, and do the data support the conclusions?

The manuscript must describe a technically sound piece of scientific research with data that supports the conclusions. Experiments must have been conducted rigorously, with appropriate controls, replication, and sample sizes. The conclusions must be drawn appropriately based on the data presented.

Reviewer #1: Yes

Reviewer #2: Yes

**********

3. Has the statistical analysis been performed appropriately and rigorously?

Reviewer #1: Yes

Reviewer #2: Yes

**********

4. Have the authors made all data underlying the findings in their manuscript fully available?

The PLOS Data policy requires authors to make all data underlying the findings described in their manuscript fully available without restriction, with rare exception (please refer to the Data Availability Statement in the manuscript PDF file). The data should be provided as part of the manuscript or its supporting information, or deposited to a public repository. For example, in addition to summary statistics, the data points behind means, medians and variance measures should be available. If there are restrictions on publicly sharing data—e.g. participant privacy or use of data from a third party—those must be specified.

Reviewer #1: Yes

Reviewer #2: Yes

**********

5. Is the manuscript presented in an intelligible fashion and written in standard English?

PLOS ONE does not copyedit accepted manuscripts, so the language in submitted articles must be clear, correct, and unambiguous. Any typographical or grammatical errors should be corrected at revision, so please note any specific errors here.

Reviewer #1: Yes

Reviewer #2: Yes

**********

6. Review Comments to the Author

Please use the space provided to explain your answers to the questions above. You may also include additional comments for the author, including concerns about dual publication, research ethics, or publication ethics. (Please upload your review as an attachment if it exceeds 20,000 characters)

Reviewer #1: After thoroughly reviewing the revisions by the authors of manuscript PONE-D-26-06317R1, titled "A polymorphism and expression levels of SCD and their relationships to production traits in a Japanese farmed emu population," and evaluating it against PLOS One's acceptance criteria, I present the following comments:

1. All improvement suggestions from reviewers 1 and 2 have been successfully implemented and thoroughly explained, leading to a clearer and more concise discussion.

2. Overall, the paper appears to be the author's original research, with no evidence of prior publication.

3. Following enhancements to the research methodology, the statistics and analysis are now clearer, enabling a thorough discussion of the results.

4. The conclusions have been revised to highlight the findings and remove any speculative elements unrelated to the research.

5. Overall, the paper is composed in standard English.

6. The study involved animals and followed procedures aligned with ethical standards for experimentation and research integrity.

7. The article adheres to relevant reporting guidelines and community standards regarding data availability.

After reviewing the revised manuscript, I find that this research offers valuable new insights into the under-studied field of emu research. Hence, I recommend the paper for publication in PLOS One.

Reviewer #2: Authors made the necessary amendments and the article is substantially improved. I suggest its acceptance

**********

7. PLOS authors have the option to publish the peer review history of their article (what does this mean?). If published, this will include your full peer review and any attached files.

If you choose “no”, your identity will remain anonymous but your review may still be made public.

**Do you want your identity to be public for this peer review?**  For information about this choice, including consent withdrawal, please see our Privacy Policy.

Reviewer #1: **Yes:** Ronny Rachman Noor

Reviewer #2: No

**********

# Editor report: Acceptance letter

**DOI:** 10.1371/journal.pone.0352695.r004

**Authors:** Abbas Farmany

PONE-D-26-06317R1

PLOS One

Dear Dr. Wada,

I'm pleased to inform you that your manuscript has been deemed suitable for publication in PLOS One. Congratulations! Your manuscript is now being handed over to our production team.

At this stage, our production department will prepare your paper for publication. This includes ensuring the following:

* All references, tables, and figures are properly cited

* All relevant supporting information is included in the manuscript submission,

* There are no issues that prevent the paper from being properly typeset

You will receive further instructions from the production team, including instructions on how to review your proof when it is ready. Please keep in mind that we are working through a large volume of accepted articles, so please give us a few days to review your paper and let you know the next and final steps.

Lastly, if your institution or institutions have a press office, please let them know about your upcoming paper now to help maximize its impact. If they'll be preparing press materials, please inform our press team within the next 48 hours. Your manuscript will remain under strict press embargo until 2 pm Eastern Time on the date of publication. For more information, please contact onepress@plos.org.

You will receive an invoice from PLOS for your publication fee after your manuscript has reached the completed accept phase. If you receive an email requesting payment before acceptance or for any other service, this may be a phishing scheme. Learn how to identify phishing emails and protect your accounts at https://explore.plos.org/phishing.

If we can help with anything else, please email us at customercare@plos.org.

Thank you for submitting your work to PLOS ONE and supporting open access.

Kind regards,

PLOS ONE Editorial Office Staff

on behalf of

Dr. Abbas Farmany

Academic Editor

PLOS One
